# Magnetic-biased chiral molecules enabling highly oriented photovoltaic perovskites

**DOI:** 10.1093/nsr/nwad305

**Published:** 2023-12-08

**Authors:** Jing Chen, Caner Deger, Zhen-Huang Su, Kai-Li Wang, Guang-Peng Zhu, Jun-Jie Wu, Bing-Chen He, Chun-Hao Chen, Tao Wang, Xing-Yu Gao, Ilhan Yavuz, Yan-Hui Lou, Zhao-Kui Wang, Liang-Sheng Liao

**Affiliations:** Institute of Functional Nano & Soft Materials (FUNSOM), Laboratory of Advanced Negative Carbon Technologies, Soochow University, Suzhou 215123, China; Department of Physics, Marmara University, Ziverbey 34722, Turkey; Shanghai Synchrotron Radiation Facility (SSRF), Shanghai Advanced Research Institute, Chinese Academy of Sciences, Shanghai 201204, China; Institute of Functional Nano & Soft Materials (FUNSOM), Laboratory of Advanced Negative Carbon Technologies, Soochow University, Suzhou 215123, China; Institute of Functional Nano & Soft Materials (FUNSOM), Laboratory of Advanced Negative Carbon Technologies, Soochow University, Suzhou 215123, China; Institute of Functional Nano & Soft Materials (FUNSOM), Laboratory of Advanced Negative Carbon Technologies, Soochow University, Suzhou 215123, China; Shanghai Synchrotron Radiation Facility (SSRF), Shanghai Advanced Research Institute, Chinese Academy of Sciences, Shanghai 201204, China; Institute of Functional Nano & Soft Materials (FUNSOM), Laboratory of Advanced Negative Carbon Technologies, Soochow University, Suzhou 215123, China; Institute of Functional Nano & Soft Materials (FUNSOM), Laboratory of Advanced Negative Carbon Technologies, Soochow University, Suzhou 215123, China; Shanghai Synchrotron Radiation Facility (SSRF), Shanghai Advanced Research Institute, Chinese Academy of Sciences, Shanghai 201204, China; Department of Physics, Marmara University, Ziverbey 34722, Turkey; College of Energy, Soochow Institute for Energy and Materials Innovations, Soochow University, Suzhou 215006, China; Institute of Functional Nano & Soft Materials (FUNSOM), Laboratory of Advanced Negative Carbon Technologies, Soochow University, Suzhou 215123, China; Institute of Functional Nano & Soft Materials (FUNSOM), Laboratory of Advanced Negative Carbon Technologies, Soochow University, Suzhou 215123, China; Macao Institute of Materials Science and Engineering, Macau University of Science and Technology, Macau, China

**Keywords:** perovskite photovoltaics, magnetic moment, A-site regulation, chiral molecules

## Abstract

The interaction between sites A, B and X with passivation molecules is restricted when the conventional passivation strategy is applied in perovskite (ABX_3_) photovoltaics. Fortunately, the revolving A-site presents an opportunity to strengthen this interaction by utilizing an external field. Herein, we propose a novel approach to achieving an ordered magnetic dipole moment, which is regulated by a magnetic field via the coupling effect between the chiral passivation molecule and the A-site (formamidine ion) in perovskites. This strategy can increase the molecular interaction energy by approximately four times and ensure a well-ordered molecular arrangement. The quality of the deposited perovskite film is significantly optimized with inhibited nonradiative recombination. It manages to reduce the open-circuit voltage loss of photovoltaic devices to 360 mV and increase the power conversion efficiency to 25.22%. This finding provides a new insight into the exploration of A-sites in perovskites and offers a novel route to improving the device performance of perovskite photovoltaics.

## Teaser

The crystal orientation of metal halide perovskites can be proficiently modulated through the magnetic dipole moments generated by chiral molecules interacting with the perovskites, regulating by an external magnetic field.

## INTRODUCTION

Metal halide perovskite solar cells (PSCs) have garnered considerable attention in the field of photovoltaics due to their remarkable and rapid advancements [1–5]. Over a span of just over a decade, their certified power conversion efficiency (PCE) has surged to 26.1%, approaching the upper limit observed in crystalline silicon cells [6]. Moreover, PSCs still hold immense potential to achieve PCEs exceeding 30% or even surpass their theoretical limitation. The optimization of cell parameters in photovoltaic devices heavily relies on the deposition of high-quality perovskite films [7–11]. To enhance the device performance, it is crucial to attain perovskite films with minimal defect density and excellent homogeneity. A commonly employed strategy involves the introduction of specific molecules during perovskite film crystallization or recrystallization, aiming to eliminate defects or prevent excessive components [12–16]. However, the conventional passivation strategies adopted in perovskite (ABX_3_) photovoltaics imposes limitations on the interaction between the passivation molecules and sites A, B and X.

Generally, the lead–iodine octahedral structure serves as the fundamental framework for governing the regulation of perovskite crystallization. The manipulation of perovskite crystallization can be achieved through the incorporation of appropriate functional groups, thereby facilitating the formation of uniform polycrystalline films with exceptional crystal orientation [17–23]. The distinctive molecular configuration exhibited by perovskites presents an opportunity to affect the crystallization orientation by modulating the polarity between the perovskite material and the introduced components. For instance, the rotation motion of the A-site offers a means to strengthen the interaction between passivation molecules and perovskites, particularly in the presence of an external field.

Herein, we developed a novel approach to effectively manipulate the crystal orientation of metal halide perovskites by introducing a series of magnetic-biased amino acid chiral molecules. By leveraging the inherent chiral characteristics, a quantifiable magnetic dipole moment is established between the amino acid molecules and perovskites. Furthermore, the external magnetic field enables a powerful enhancement of the spin–orbit coupling effect between formamidine (FA) ions and chiral molecules, thereby facilitating precise regulation of crystal orientation in perovskite films. Through this strategy, the external magnetic field and the internal magnetic dipole moment cooperate with each other to effectively regulate and optimize the crystallization of perovskite films. In comparison with the conventional passivation strategy involving direct implementation of organic molecules, our proposed method demonstrated a remarkable enhancement in the interaction energy (Δ*E*) between chiral molecules and perovskites, achieving an ∼4-fold increase. This substantial improvement ensures effective crystal regulation and guarantees the ordered arrangement of passivation molecules on the surface of perovskite films. Consequently, the quality of the perovskite film was significantly enhanced, resulting in a notable suppression of nonradiative recombination. The fabricated photovoltaic devices delivered a promising PCE of 25.22% (compared with 23.72% for the control device) by reducing the loss of open-circuit voltage to 360 mV. Furthermore, the stability of the unpackaged devices was successfully preserved, as shown by the retention of 94.2% of their original efficiency following a 967-hour steady-state power production at maximum power point (MPP).

## THEORETICAL EXPECTATION AND EXPERIMENTAL SUPPORT

**Figure 1.**
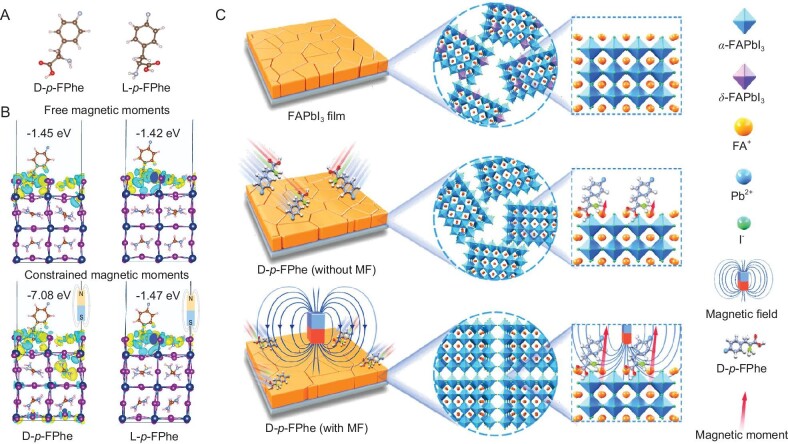
(A) Molecular formula for the D/L-*p*-fluorophenylalanine molecules. (B) The charge density profiles and binding energies for the D/L-*p*-FPhe molecules on the perovskite surface with or without a magnetic field. (C) Schematic illustration of the comparison of the crystal structure inside the pristine perovskite film, D-*p*-FPhe-doped film and D-*p*-FPhe-doped film with an applied magnetic field.

The chiral molecules used in this study, namely *p*-fluorophenylalanine (*p*-FPhe) and its learned (L) and dexter (D) configurations, were selected as illustrated in Fig. 1A. Density functional theory (DFT) simulations were conducted to confirm that both the chiral molecules and the perovskite structure lacked any magnetic dipole moments (Supplementary Figs S1 and S2). Previous theoretical studies have demonstrated that a rearrangement of chiral molecules can induce electric and magnetic dipole moments, resulting in a current at the end of the molecular ring [24–26]. To investigate the effect of intrinsic dipole moments of chiral molecules on their binding interactions with perovskites, we examined the situation of chiral molecules bound to the perovskite surface. The DFT results demonstrated a specific spin–orbit coupling between chiral molecules and moderately dynamic FA cations (Supplementary Fig. S3). Compared with previous reports, chiral molecules tend to passivate charged defects such as vacancies and uncoordinated sites, and the consequences of such coupling are frequently subtle and imperceptible [27].

To gain a deep understanding of the underlying mechanism, we conducted modeling to investigate the impact of an external magnetic field on the magnetic moments. The binding energies and charge distributions between D/L-*p*-FPhe and the perovskite surface were calculated in both the absence and presence of a magnetic field. As depicted in Fig. 1B, without a magnetic field, the binding energies and charge density distributions of D/L-*p*-FPhe molecules and perovskites exhibited comparable characteristics. In contrast, upon introducing a magnetic field, the binding energy between D-*p*-FPhe and perovskite increased to 7.08 eV. The difference in molecular interaction energy $\Delta E$ (${E}_2 - {E}_1$, where ${E}_2$ and ${E}_1$ represent the binding energies of chiral molecules attached to the perovskites with or without a magnetic field) increased by 4-fold, while L-*p*-FPhe showed only a slight difference (1.47 eV). This result was attributed to the enhanced spin–orbit coupling, which resulted in a more efficient and compact interaction. Additionally, D-*p*-FPhe induced considerable charge transfer along the *z*-axis, as illustrated in Supplementary Fig. S4, resulting in a notable alteration in the charge density distribution within the perovskite structure. This indicates that an external magnetic field has the potential to increase the magnetic moment and strengthen the coupling effect. However, no similar behavior was observed for the L-*p*-FPhe molecule. The D-*p*-FPhe molecule tended to align vertically (87.5°) on the surface and its initial magnetic dipole moment was stronger than that of the L-*p*-FPhe molecule (Supplementary Fig. S5), which might be related to the distance between the benzene ring and the acting sites. The angle between the molecule and the perovskite structure suggested a difference in electron density distribution and strength of interaction. A magnetic field will further amplify the alteration in the electron cloud distribution, resulting in differentiation in the manifestation of magnetic dipole moments. In summary, the existence of a distinct and strong spin–orbit coupling effect compared with the coordination effects between functional groups and perovskites was confirmed. By harnessing magnetic dipole moments and magnetic fields, it is possible to effectively control intrinsic properties such as the charge density distribution of perovskites.

This study focused on planar heterojunction PSCs with a device structure of SnO_2_: F (FTO)/tin oxide (SnO_2_)/CH(NH_2_)_2_PbI_3_ (FAPbI_3_)/Spiro-OMeTAD/Au. As depicted in Fig. 1C, due to the large size of the FA ions, the polycrystalline perovskite film formed via a one-step spin-coating process inevitably contains a mixture of *α* and *δ* phases. Nevertheless, the black phase $\alpha $ with a reduced band gap is what we truly desire [28,29]. Here, we present a single amino acid molecule for defect passivation. In particular, the focus in this study is on the magnetic dipole moment, which is a distinctive characteristic of this material. This interaction can cause the generation of quantitatively oriented magnetic dipole moments at specific sites. Consequently, the distribution and polarity of electrons are altered, ultimately guiding the crystallization orientation of perovskite films. To further enhance this induction (crystallization process), an external magnetic field was applied during the spin-coating process. An appropriate magnetic field (MF) can amplify the spin–orbit coupling effect between FA ions and chiral molecules, significantly increasing the magnetic dipole moment and effectively controlling the crystallization process. This strategic approach can improve crystallization and promote orientation homogenization, resulting in enhanced charge transport and improved device performance.

The feasibility of the proposed strategy was assessed through computational calculations, which provided theoretical evidence for its viability. To further validate the specific effects of the strategy, a series of measurements were conducted to investigate the interactions involved. The X-ray photoelectron spectroscopy (XPS) curves of D-*p*-FPhe-doped films subjected to the application of a magnetic field (referred to as the target condition) exhibited an anticipated modification in the distribution of electron densities on the surface of the film. The control samples indicated no passivation and no magnetic field influences, the L-*p*-FPhe samples indicated the passivation condition (with functional groups but without the influence of the magnetic field) and the D-*p*-FPhe samples indicated the results of the magnetic field (with magnetic dipole moment but almost without passivation influence), according to the theoretical results derived from Fig. 1. In the absence of more explanation, the following sample conditions are likewise provided as detailed here.

**Figure 2.**
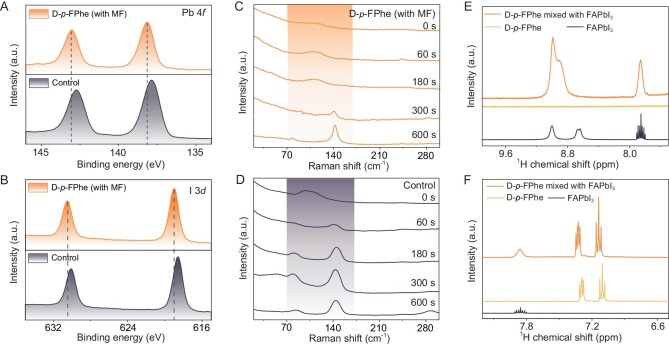
XPS spectra of (A) Pb 4f peaks and (B) I 3d peaks for perovskite film doped with D-*p*-FPhe under a magnetic field and control FAPbI_3_ perovskite film. Raman spectra of (C) perovskite film-doped D-*p*-FPhe under a magnetic field and (D) the control FAPbI_3_ perovskite film recorded at room temperature. Liquid-state 1H NMR spectra of FAPbI_3_, D-*p*-FPhe and FAPbI_3_/D-*p*-FPhe mixed powders dissolved in DMSO-d_6_ for the (E) NH_2_ group region and (F) benzene ring region.

Figure 2A and B illustrates the observed tendencies in the electron density distribution within the [PbI_6_]^4−^ octahedral structure under the influence of the magnetic dipole moment. While a weaker influence was supported by the smaller shift of the Pb 4*f* peaks and the I 3*d* XPS patterns (Supplementary Fig. S6) for the L-*p*-FPhe samples, the core level peaks of Pb 4*f* and I 3*d* were observed to shift towards higher energy levels, indicating the migration and accumulation of electrons near the A-sites due to the presence of the magnetic dipole moment. This evidence underscores the reduced impact of the L-*p*-FPhe molecule on the charge distribution when compared with its D-*p*-FPhe counterpart. These experimental findings aligned with the estimated outcomes and provided further confirmation.

To comprehensively investigate the interaction between D-*p*-FPhe and FA ions, Raman spectroscopy was employed to monitor the crystallization process of the film. Figure 2C and D depicts the Raman spectra of the perovskite film, with a focus on the high-coupling range (50–200 cm^−1^) [30]. During the early stages of the reaction, the insertion of FA ions into the crystal lattice was observed, as evidenced by the appearance of peaks at 140 cm^−1^ [31]. As the crystal expanded, the intensity of these peaks gradually increased, ultimately reaching 140 cm^−1^. This observation indicated that the crystal formation process of the target film proceeded at a slower rate compared with the control sample (a pure perovskite film without the application of a magnetic field). In contrast, the control sample exhibited an earlier appearance of the perovskite phase and a stronger Raman signal associated with twist and distortion vibrations, characteristic of octahedral modes within the range of 50–80 cm^−1^ [32]. Notably, the signals associated with PbI_2_ (∼80 cm^−1^) due to *δ* phases were significantly reduced in the target films, while the *α* phase signal was smaller and more concentrated at ∼144–140 cm^−1^ [33]. Additionally, discernible differences between the two samples were observed at ∼300 cm^−1^, corresponding to the ‘symmetric and asymmetric out-of-plane bending of FA ions’ modes [33].

The utilization of our strategy facilitated the disappearance of *δ* phase signals in a faster and more comprehensive manner, resulting in stronger and narrower α phase signals. Differently from the traditional perovskite crystallization strategies such as the MACl (methylammonium chloride)-induced method, which work by introducing molecules into the crystallization process, altering local ion concentrations to influence phase transition temperatures, optimizing the resulting phases from a chemical perspective, the magnetic dipole moment tends to perform as an attractive force inside the perovskite structure. This effect is much stronger than traditional chemical interactions, thus the crystallization quality can be controlled more effectively and adequately [34,18]. These concurrent findings indicated that the magnetic dipole moment exerted control over the FA ions during the crystallization process, enhancing their behavior. Furthermore, the coupling conditions between the FA ions and the chiral molecules were corroborated by the results obtained from nuclear magnetic resonance (NMR) analysis. Figure 2E displays the region corresponding to the NH_2_ group signal of the FA ions in perovskites. Initially, the 1H–14N J-coupling frequency exhibited disparities due to the (partial) diamagnetic anisotropy associated with the double bond [35]. Upon interaction with chiral compounds, the proton signal peak with greater J-coupling experienced a significant shift (0.22 ppm) towards a lower field, indicating changes in the vicinal trans coupling with the C–H proton in FA ions. These results confirmed the effective coupling between the chiral molecules and FA ions. Figure 2F illustrates the H signals corresponding to the benzene ring. The primary coupling site was identified between the FA ions and the NH_2_ group of the chiral molecules, which aligns well with the computational predictions. Although there were changes in the electron cloud distribution on the benzene ring of the chiral molecule upon combination, the chemical shift remained minimal.

## MAGNETIC MOMENT-INDUCED CRYSTAL REGULATION

**Figure 3.**
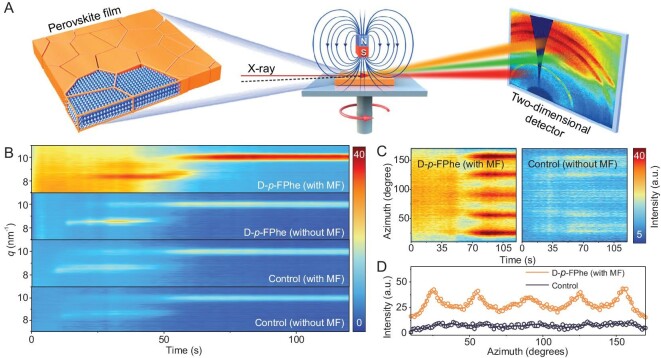
(A) Schematics of the set-up for the perovskite film preparation with magnetic field via *in situ* GIWAXS. (B) *In situ* GIWAXS monitoring of the crystal growth process of perovskite films. Top-view image of FAPbI_3_ (doped with D-*p*-FPhe) (001) peaks with/without a magnetic field in GIWAXS images. (C) 2D plots of the azimuthally integrated scattering intensity varying with annealing time along the ring at *q* = 10 nm^−1^ for D-*p*-FPhe (with MF) films and control films. (D) The corresponding radially integrated intensity plots along the ring at *q*= 10 nm^−1^ for the two films derived from Fig. 3C.

*In situ* grazing-incidence wide-angle X-ray scattering (GIWAXS) offers valuable insights into the regulatory influences on the crystal growth process and the resultant quality of the final polycrystalline film. To investigate the perovskite crystal growth process, a ‘one-step antisolvent’ preparation method was employed under controlled laboratory conditions, as depicted in Fig. 3A. The process was conducted in a nitrogen atmosphere, while a magnetic field of specific intensity was applied by manipulating the distance between the rubidium iron–boron (NdFeB) magnet and the perovskite films.

Our investigation focused on comprehending the phase changes that occur during the complete crystallization of perovskite films. The emergence of an intermediate perovskite phase was observed upon the addition of antisolvents (at 15 s) and the crystallization process reached completion within ∼90 s. Figure 3B demonstrates that D-*p*-FPhe molecules exhibited enhanced passivation properties in the absence of a magnetic field, thereby optimizing the crystallization process. Prior studies have reported marginal improvements in crystallization when a magnetic field is applied [36,37]. However, our findings indicated that the application of a magnetic field significantly intensified the crystallization process in perovskite films, surpassing the effects of passivation (Supplementary Fig. S7). Furthermore, by analysing the corresponding (001) peak-intensity-related-with-time curve (Supplementary Fig. S8), it was evident that this regulation notably prolonged the duration of crystallization growth. Raman analysis, in conjunction with these observations, indicated that the insertion of FA ions into the lattice occurred at a slower rate under the influence of the magnetic dipole moment. Consequently, the crystal lattice exhibited improved orderliness, leading to enhanced crystallinity (Supplementary Fig. S9). 2D GIWAXS images of key points in the crystallization process (Supplementary Figs S10–S12) highlighted the substantial enhancement of each crystal face in the target film, resulting in clearer and sharper scattering rings. By comparing the 2D plots of the azimuth integral scattering intensity for the ring at *q*= 10 nm^−1^ as shown in Fig. 3C and its corresponding integral curves in Fig. 3D, the impact of our strategy on the crystal growth orientation across the entire film was further discerned.

The integral curves depicted in Fig. 3D provided a direct comparison of the crystallization orientations. The (001) crystal plane (*q* = 10 nm^−1^) of both films exhibited preferential multi-order orientations, characterized by peaks centered at 25°, 55° and 90°. However, the target film displayed significantly stronger and narrower intensity as well as a reduced full width at half maximum compared with the control film. This result suggested that the structure of the target film was better organized and uniform, indicative of enhanced crystalline quality and uniformity [38,39]. To gain further insights into the phase uniformity (anisotropy) contributed by this strategy, polar photoluminescence (PL) images of the crystallization process were captured using a micro zone PL microscope. Anisotropy arises from variations in light absorption at different polarization angles, which in turn stem from differences in the refractive index along various crystal axes [40]. In comparison with the control samples, as demonstrated in Supplementary Figs S13 and S14, the target samples exhibited a more rapid development of a bimodal shape at a fixed position during growth, leading to sharper and better symmetry in the final image. Supplementary Fig. S15 illustrates that the control samples exhibited weaker polarization dependence, with the corresponding degree of polarization increasing from 0.05 to 0.10 after optimization [41]. This preferential phase orientation facilitated carrier transfer along the favored orientations and promoted carrier recombination [42,43].

**Figure 4.**
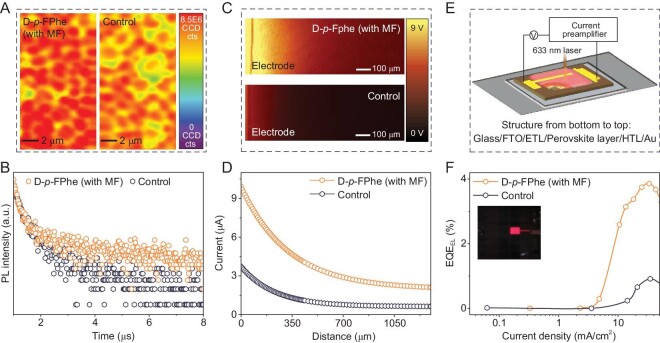
(A) PL mapping of D-*p*-FPhe (with MF) and control films. (B) TRPL spectra of D-*p*-FPhe (with MF) and control films. (C) Photocurrent mapping of D-*p*-FPhe (with MF) and control devices. (D) SPMS spectra of D-*p*-FPhe (with MF) and control devices extracted from Fig. 4C. (E) Schematic illustration of the set-up for the SPMS measurement. (F) EL EQE of D-*p*-FPhe (with MF) and control devices; the inset shows the EL picture.

Stable-state photoluminescence (PL) was obtained to analyse the carrier dynamics. Supplementary Fig. S16 displays a noticeable enhancement in the PL intensity of the target film compared with the control film, indicating a significant reduction in defect density within the target films. To further investigate the quality of the perovskite films at the nanoscale, confocal fluorescence images were obtained, as depicted in Fig. 4A. Spatial variations in PL intensity often serve as indicators of local nonradiative recombination rates. Consequently, the greater and more uniform PL intensity observed in the target sample provided visual evidence that the induction of a magnetic dipole moment substantially improved the quality of the polycrystalline film while simultaneously reducing nonradiative recombination sites. The time-resolved photoluminescence (TRPL) results are presented in Fig. 4B and the related statics are listed in Supplementary Table S1. Both the target and control samples exhibited two decay states. Considering the rapid decay process resulting from carrier diffusion in the early stage, the target film demonstrated a significantly slower decay with a *τ*_1_ value of 220 ns (compared with 90 ns for the control film). This slower decay was primarily attributed to the reduction in defect density within the target film [43]. In the slow single-exponential decay process associated with Shockley–Read–Hall recombination and surface recombination, the target film exhibited an effectively extended lifetime (3.60 μs), which was much longer than that of the control film (0.42 μs). This prolonged lifetime reflected the successful elimination of electron trap states facilitated by the magnetic dipole moment [44].

Upon establishing the positive carrier optimization effect in thin films, our investigation shifted towards understanding the impact of this optimization on device functionality. To gain insights into surface carrier transport in action, we conducted photocurrent measurements on both the target and the control devices. The scanning photocurrent measurement system (SPMS) allowed us to acquire photocurrent data at a location that was relatively distant from the electrode edge of the devices. In Fig. 4C, the dark line represents the boundary between the gold electrode and the perovskite region. Notably, the target device exhibited a significantly stronger photocurrent signal, covering a larger area compared with the control device. This observation suggested that the high-quality perovskite layer possessed superior photon absorption and carrier conversion capabilities. The absorption spectra further confirmed the better absorption ability of the target films (Supplementary Fig. S17).

We further conducted a fitting analysis on the photocurrent intensity and distance curves, as depicted in Fig. 4D, to provide a more accurate and intuitive comparison of photogenerated carrier transmission between the two samples (related values are provided in Supplementary Table S2). By fitting the curves, we determined the average carrier diffusion lengths, denoted as *l*, to be 325.46 and 230.34 μm for the target and control devices, respectively. The substantially longer diffusion length observed in the target device indicated a significant improvement in the quality of the polycrystalline film. This result suggested that the target perovskite film exhibited better order and uniformity, leading to a better charge transport ability [45]. The effective improvement of the photoelectric film quality, particularly in terms of the open-circuit voltage (*V*_oc_), would greatly enhance the performance of the device, as elucidated below.

A schematic representation of the SPMS measuring set-up can be found in Fig. 4E. The loss of *V*_oc_ in the target devices was significantly reduced by employing perovskite films in which nonradiative recombination was effectively suppressed. We employed detailed equilibrium theory, which is well known for providing thorough analysis [46,47]. The absorption spectra (Supplementary Fig. S17) and external quantum efficiency (EQE) spectra (Supplementary Fig. S18) revealed that the loss of *V*_oc_ was not closely related to Type I loss (Δ*V*_1_) and Type II loss (Δ*V*_2_). Energy band alignment obtained from UPS spectra is offered in Supplementary Figs S19 and S20 [48,49]. The last and most important part was the loss resulting from nonradiative recombination triggered by the presence of trap states (Δ*V*_3_), which could be calculated by the EQE obtained by using the electroluminescence (EL) test. Figure 4F plots the EL_EQE_ curves of the target and control devices [50]. The inset diagram shows the EL image taken while the target devices functioned as light-emitting diodes (LEDs) (Supplementary Fig. S21). With an injection current of 26 mA/cm^2^ (roughly equivalent to the short-circuit current density, *J*_sc_), the EL efficiency of the target device reached 3.81%, effectively reducing the loss from 126.82 to 86.26 mV. The overall *V*_oc_ aligned well with the actual values as offered in Supplementary Table S4, resulting in a notable enhancement of the device performance. Further elaboration on the device performance will be provided in the subsequent section.

## PHOTOELECTRIC PERFORMANCE AND STABILITY

**Figure 5.**
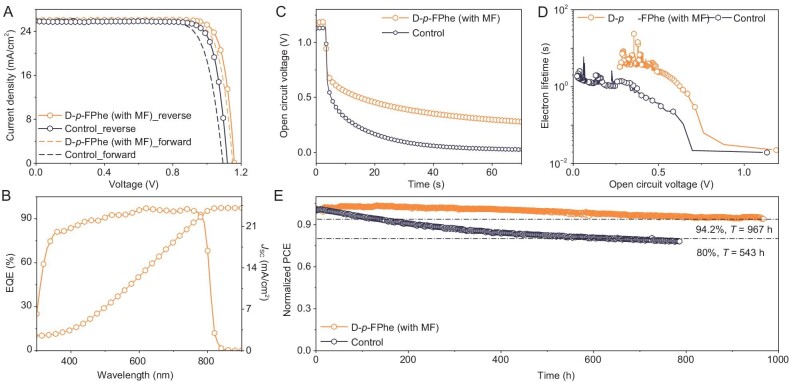
(A) *J–V* curves of D-*p*-FPhe (MF) and control devices, measured in reverse (solid line) and forward (dashed line) modes. (B) EQE diagram and corresponding integral current of the D-*p*-FPhe (MF) device. (C) OCVD curves of D-*p*-FPhe (MF) and control devices. (D) Electron lifetime of the D-*p*-FPhe (MF) and control devices extracted from OCVD measurement. (E) Stabilized maximum power point (MPP) values record of the devices for 967 hours without encapsulation under continuous light irradiation using a white LED lamp at 100 mW cm^–2^ in a nitrogen environment.

The control and target photovoltaic devices were fabricated and characterized as shown in Fig. 5A and a comprehensive summary of their performance parameters is presented in Supplementary Table S3. The developed strategy enabled a remarkable enhancement of the PCE, increasing it from 23.72% to 25.22%. The improvement was primarily attributed to the rise in *V*_oc_. The corresponding EQE curve and integrated *J*_sc_ in the target device are plotted in Fig. 5B (the control device is shown in Supplementary Fig. S22). To gain a better understanding of the impact of magnetic dipole moment induction on charge recombination in operational devices, open-circuit photovoltage decay (OCVD) tests were conducted. As shown in Fig. 5C, the initial photovoltage of the target device was 1.16 V, which was higher than the 1.12 V of the control device. The decay of the photovoltage was observed to be delayed and the corresponding electron lifetime was further extracted to obtain Fig. 5D. It was evident that the electron lifetime in the target device was effectively prolonged and consistently exceeded that of the control device, which was in agreement with the SPMS result. Energy and depth distributions of the trap densities were further evaluated by using thermal admittance spectroscopy and drive-level capacitance profiling (DLCP). The trap density of states (*t*DOS) curves (Supplementary Fig. S23) and DLCP curves (Supplementary Fig. S24) demonstrated that the defects in the target device were largely suppressed. This implied that our strategy could successfully improve the overall quality of the perovskite film. A total of 32 control and target devices were fabricated and tested, with the target devices exhibiting a predominant distribution around a PCE of 24.5% (Supplementary Fig. S25). This observation indicated good reproducibility of the fabrication process. We conducted the stabilized MPP values record of the devices for ∼1000 hours (967 hours) without encapsulation under continuous light irradiation with a white LED lamp at 100 mW cm^–2^ in a nitrogen environment as shown in Fig. 5E. It presents that the target devices had an excellent stability, maintaining 94.2% of the initial value after ∼1000 hours, while the control devices dropped to <80% after 543 hours. The storage stability also exhibited the same results as presented in Supplementary Fig. S26. The unpackaged control and target devices were subjected to stability testing by storing them in dry air at room temperature. After 4200 hours of aging, the control device experienced a decline to <60% of its initial efficiency, whereas the target device could still maintain 90% of the initial value. These findings conclusively demonstrated a positive role of the new strategy in improving device stability.

## CONCLUSION

In summary, we have demonstrated a unique interaction termed a magnetic dipole moment between the perovskite and the chiral molecule, which can be further enhanced by using an external magnetic field. By appropriately inducing the magnetic dipole moment, the interaction energy experienced a substantial improvement, reaching a 4-fold increase. The enlarged interaction facilitated facile regulation of crystal orientation and ensured the ordered arrangement of passivation molecules on the surface of perovskite films. Consequently, the quality of the perovskite film was greatly enhanced, resulting in a remarkable suppression of nonradiative recombination. Owing to a large reduction in *V*_oc_ loss to 360 mV, the target device presented a promising PCE as high as 25.22%. Furthermore, the stability of the unpackaged devices was effectively maintained, as demonstrated by the preservation of 94.2% of the initial efficiency after undergoing a 967-hour steady-state power output at MPP.

Looking towards the future, there remains considerable unexplored potential in investigating the magnetic-dependent properties of chiral molecules in perovskite optoelectronic devices. For instance, by carefully selecting functional groups characterized by greater asymmetry or stronger binding affinity, it is possible to further enhance the regulatory capabilities of the magnetic dipole moment. It is also advisable to opt for those containing benzene rings to enhance their optical activity, which is somewhat related to the intrinsic magnetic dipole moment of the chiral molecules themselves. Increasing the number of fluorine substitutions to enhance resistance to annealing temperature allows the possibility of promoting this strategy. This study offers a novel avenue for precisely controlling the crystal quality of perovskite films with a target towards realizing high-performance perovskite optoelectronic devices.

## Supplementary Material

nwad305_Supplemental_File

## References

[1] Kim HS , Lee CR, Im JH et al. Lead iodide perovskite sensitized all-solid-state submicron thin film mesoscopic solar cell with efficiency exceeding 9%. Sci Rep 2012; 2: 591. 10.1038/srep00591 22912919 PMC3423636

[2] Burschka J , Pellet N, Moon SJ et al. Sequential deposition as a route to high-performance perovskite-sensitized solar cells. Nature 2013; 499: 316–9. 10.1038/nature12340 23842493

[3] Zhao Y , Zhu PC, Wang MH et al. A polymerization-assisted grain growth strategy for efficient and stable perovskite solar cells. Adv Mater 2020; 32: 1907769. 10.1002/adma.201907769 32147861

[4] Senanayak SP , Abdi-Jalebi M, Kamboj VS et al. A general approach for hysteresis-free, operationally stable metal halide perovskite field-effect transistors. Sci Adv 2020; 6: eaaz4948. 10.1126/sciadv.aaz4948 32300658 PMC7148112

[5] Wehrenfennig C , Eperon GE, Johnston MB et al. High charge carrier mobilities and lifetimes in organolead trihalide perovskites. Adv Mater 2014; 26: 1584–9. 10.1002/adma.201305172 24757716 PMC4722848

[6] NREL . Best Research-Cell Efficiencies. https://www.nrel.gov/pv/assets/images/cell-pv-eff-emergingpv.png (23 November 2023, data last accessed).

[7] Ma J , Qin MC, Li YH et al. Unraveling the impact of halide mixing on crystallization and phase evolution in CsPbX_3_ perovskite solar cells. Matter 2021; 4: 313–27. 10.1016/j.matt.2020.10.023

[8] Li L , Chen Y, Wang X et al. The additive coordination effect on hybrids perovskite crystallization and high-performance solar cell. Adv Mater 2016; 28: 9862–8. 10.1002/adma.201603021 27709662

[9] Zheng X , Deng YH, Chen B et al. Dual functions of crystallization control and defect passivation enabled by sulfonic zwitterions for stable and efficient perovskite solar cells. Adv Mater 2018; 30: 1803428. 10.1002/adma.201803428 30370954

[10] Xiong Z , Chen X, Zhang B et al. Simultaneous interfacial modification and crystallization control by biguanide hydrochloride for stable perovskite solar cells with PCE of 24.4%. Adv Mater 2022; 34: 2106118. 10.1002/adma.202106118 34862820

[11] Li Y , Chen ZJ, Yu BC et al. Efficient, stable formamidinium-cesium perovskite solar cells and minimodules enabled by crystallization regulation. Joule 2022; 6: 676–89. 10.1016/j.joule.2022.02.003

[12] Luo D , Yang WQ, Wang ZP et al. Enhanced photovoltage for inverted planar heterojunction perovskite solar cells. Science 2018; 360: 1442–6. 10.1126/science.aap9282 29954975

[13] Xue J , Wang R, Wang KL et al. Crystalline liquid-like behavior: surface-induced secondary grain growth of photovoltaic perovskite thin film. J Am Chem Soc 2019; 141: 13948–53. 10.1021/jacs.9b06940 31403287

[14] Wang J , Luo SQ, Lin Y et al. Templated growth of oriented layered hybrid perovskites on 3D-like perovskites. Nat Commun 2020; 11: 582. 10.1038/s41467-019-13856-1 31996680 PMC6989653

[15] Zhao Y , Ma F, Qu ZH et al. Inactive (PbI_2_)_2_RbCl stabilizes perovskite films for efficient solar cells. Science 2022; 377: 531–4. 10.1126/science.abp8873 35901131

[16] Jiang Q , Tong JH, Xian YM et al. Surface reaction for efficient and stable inverted perovskite solar cells. Nature 2022; 611: 278–83. 10.1038/s41586-022-05268-x 36049505

[17] Wang M , Zhao Y, Jiang X et al. Rational selection of the polymeric structure for interface engineering of perovskite solar cells. Joule 2022; 6: 1032–48. 10.1016/j.joule.2022.04.002

[18] Kim M , Kim GH, Lee TK et al. Methylammonium chloride induces intermediate phase stabilization for efficient perovskite solar cells. Joule 2019; 3: 2179–92. 10.1016/j.joule.2019.06.014

[19] Han X , Wang X, Feng J et al. Carrier mobility enhancement in (121)-oriented CsPbBr_3_ perovskite films induced by the microstructure tailoring of PbBr_2_ precursor films. ACS Appl Electron Mater 2021; 3: 373–84. 10.1021/acsaelm.0c00909

[20] Zhao P , Su J, Lin Z et al. The crystal anisotropy effect of MAPbI_3_ perovskite on optoelectronic devices. Mater Today Energy 2020; 17: 100481. 10.1016/j.mtener.2020.100481

[21] Li B , Shen T, Yun S. Recent progress of crystal orientation engineering in halide perovskite photovoltaics. Mater Horiz 2023; 10: 13–40. 10.1039/D2MH00980C 36415914

[22] Lee JW , Bae YT, Hsieh YT et al. A bifunctional Lewis base additive for microscopic homogeneity in perovskite solar cells. Chem 2017; 3: 290–302. 10.1016/j.chempr.2017.05.020

[23] Bai Y , Huang Z, Zhang X et al. Initializing film homogeneity to retard phase segregation for stable perovskite solar cells. Science 2022; 378: 747–54. 10.1126/science.abn3148 36395230

[24] Freedman TB , Diem M, Polavarapu PL. Vibrational circular dichroism in amino acids and peptides. 6. Localized molecular orbital calculations of the carbon-hydrogen stretching vibrational circular dichroism in deuterated isotopomers of alanine. J Am Chem Soc 1982; 104: 3343–9. 10.1021/ja00376a016

[25] Nafie LA , Oboodi MR, Freedman TB. Vibrational circular dichroism in amino acids and peptides. 8. A chirality rule for methine C_α_-H stretching modes. J Am Chem Soc 1983; 105: 7449–50. 10.1021/ja00363a044

[26] Wang W . Electron spin and the origin of bio-homochirality. I. Extant enzymatic reaction model. arXiv:1309.1229.

[27] Yang S , Dai J, Yu Z et al. Tailoring passivation molecular structures for extremely small open-circuit voltage loss in perovskite solar cells. J Am Chem Soc 2019; 141: 5781–7. 10.1021/jacs.8b13091 30888171

[28] Fu C , Gu Z, Tang Y et al. From structural design to functional construction: amine molecules in high-performance formamidinium-based perovskite solar cells. Angew Chem 2022; 134: e202117067. 10.1002/ange.202117067 35148011

[29] Zhang T , Xu Q, Xu F et al. Spontaneous low-temperature crystallization of α-FAPbI3 for highly efficient perovskite solar cells. Sci Bull 2019; 64: 1608–16. 10.1016/j.scib.2019.08.029 36659573

[30] Driscoll E , Orera A, Anderson P. Raman spectroscopy insights into the α-and δ-phases of formamidinium lead iodide (FAPbI_3_). Dalton Trans 2021; 50: 3315–23. 10.1039/D0DT04300A 33595035

[31] Ahlawat P , Hinderhofer A, Alharbi EA et al. A combined molecular dynamics and experimental study of two-step process enabling low-temperature formation of phase-pure α-FAPbI_3_. Sci Adv 2021; 7: eabe3326. 10.1126/sciadv.abe3326 33893100 PMC8064632

[32] Ibaceta-Jaña J , Muydinov R, Rosado P et al. Vibrational dynamics in lead halide hybrid perovskites investigated by Raman spectroscopy. Phys Chem Chem Phys 2020; 22: 5604–14. 10.1039/C9CP06568G 32100759

[33] Chen S , Wang J, Ran G et al. Control of the surface disorder by ion-exchange to achieve high open-circuit voltage in HC (NH_2_)_2_PbI_3_ perovskite solar cell. Small Methods 2021; 5: 2101079. 10.1002/smtd.202101079 34928012

[34] Bi L , Fu Q, Zeng Z et al. Deciphering the roles of MA-based volatile additives for α-FAPbI_3_ to enable efficient inverted perovskite solar cells. J Am Chem Soc 2023; 145: 5920–9. 10.1021/jacs.2c13566 36877962

[35] Van Gompel WTM , Herckens R, Reekmans G et al. Degradation of the formamidinium cation and the quantification of the formamidinium–methylammonium ratio in lead iodide hybrid perovskites by nuclear magnetic resonance spectroscopy. J Phys Chem C 2018; 122: 4117–24. 10.1021/acs.jpcc.7b09805

[36] Wang H , Lei J, Gao F et al. Magnetic field-assisted perovskite film preparation for enhanced performance of solar cells. ACS Appl Mater Interfaces 2017; 9: 21756–62. 10.1021/acsami.7b03081 28589714

[37] Corpus-Mendoza AN , Moreno-Romero PM, Hu H. Impact of magnetic fields on the morphology of hybrid perovskite films for solar cells. AIP Adv 2018; 8: 055221. 10.1063/1.5026797

[38] Yang Y , Lu H, Feng S et al. Modulation of perovskite crystallization processes towards highly efficient and stable perovskite solar cells with MXene quantum dot-modified SnO_2_. Energy Environ Sci 2021; 14: 3447–54. 10.1039/D1EE00056J

[39] Yang Y , Yang L, Feng S. Interfacial engineering and film-forming mechanism of perovskite films revealed by synchrotron-based GIWAXS at SSRF for high-performance solar cells. Mater Today Adv 2020; 6: 100068. 10.1016/j.mtadv.2020.100068

[40] Han Z , Fu W, Zou Y et al. Oriented perovskite growth regulation enables sensitive broadband detection and imaging of polarized photons covering 300–1050 nm. Adv Mater 2021; 33: 2003852. 10.1002/adma.202003852 33554373

[41] Zhu H , Fu Y, Meng F et al. Lead halide perovskite nanowire lasers with low lasing thresholds and high quality factors. Nat Mater 2015; 14: 636–42. 10.1038/nmat4271 25849532

[42] Qin M , Chan PF, Lu X. A systematic review of metal halide perovskite crystallization and film formation mechanism unveiled by in situ GIWAXS. Adv Mater 2021; 33: 2105290. 10.1002/adma.202105290 34605066

[43] Liu Y , Akin S, Hinderhofer A et al. Stabilization of highly efficient and stable phase-pure FAPbI_3_ perovskite solar cells by molecularly tailored 2D-overlayers. Angew Chem Int Ed 2020; 59: 15688–94. 10.1002/anie.202005211 32400061

[44] Zhang H , Chen Z, Qin M et al. Multifunctional crosslinking-enabled strain-regulating crystallization for stable, efficient α-FAPbI_3_-based perovskite solar cells. Adv Mater 2021; 33: 2008487. 10.1002/adma.202008487 34085738

[45] Chen J , Zhu GP, Li X et al. Visualizing the surface photocurrent distribution in perovskite photovoltaics. Small 2022; 18: 2201930. 10.1002/smll.202201930 35723194

[46] Li F , Deng X, Qi F et al. Regulating surface termination for efficient inverted perovskite solar cells with greater than 23% efficiency. J Am Chem Soc 2020; 142: 20134–42. 10.1021/jacs.0c09845 33190487

[47] Qian D , Zheng Z, Yao H et al. Design rules for minimizing voltage losses in high-efficiency organic solar cells. Nat Mater 2018; 17: 703–9. 10.1038/s41563-018-0128-z 30013057

[48] Munday JN . The effect of photonic bandgap materials on the Shockley-Queisser limit. J Appl Phys 2012; 112: 064501. 10.1063/1.4742983

[49] Tvingstedt K , Malinkiewicz O, Baumann A et al. Radiative efficiency of lead iodide based perovskite solar cells. Sci Rep 2014; 4: 6071. 10.1038/srep06071 25317958 PMC5377528

[50] Yao J , Kirchaetz T, Vezie MS et al. Quantifying losses in open-circuit voltage in solution-processable solar cells. Phys Rev Applied 2015; 4: 014020. 10.1103/PhysRevApplied.4.014020

