# Supplementary material for: Magnetic-biased chiral molecules enabling highly oriented photovoltaic perovskites
Source: Natl Sci Rev. 2023 Dec 8;11(2):nwad305. doi: 10.1093/nsr/nwad305 (PMC10776365; doi:10.1093/nsr/nwad305)
Supplement: nwad305_Supplemental_File [file nwad305_supplemental_file.pdf]

Magnetic-biased Chiral Molecules Enabling Highly Orientated Photovoltaic Perovskites

Jing Chen,[1,†] Caner Deger,[2,†] Zhen-Huang Su,[3,†] Kai-Li Wang,[1] Guang-Peng Zhu,[1] Jun-Jie Wu,[1] Bing-Chen He,[3] Chun-Hao Chen,[1] Tao Wang,[1] Xing-Yu Gao,[3] Ilhan Yavuz,[2] Yan-Hui Lou,[4] Zhao-Kui Wang,[1,*] and Liang-Sheng Liao[1]

[1] Institute of Functional Nano & Soft Materials (FUNSOM), Laboratory of Advanced Negative Carbon Technologies, Soochow University, Suzhou, Soochow University, Soochow University, Suzhou 215123, China.

[2] Department of Physics, Marmara University, Ziverbey, Istanbul 34722, Turkey.

[3] Shanghai Synchrotron Radiation Facility (SSRF), Shanghai Advanced Research Institute, Chinese Academy of Sciences, Shanghai 201204, P. R. China.

[4] College of Energy, Soochow Institute for Energy and Materials Innovations, Soochow University, Suzhou 215006, China.

[5] Macao Institute of Materials Science and Engineering, Macau University of Science and Technology, Taipa 999078, Macau SAR, China.

*Corresponding Author(s): Zhao-Kui Wang and Liang-Sheng Liao

Email Address: zkwang@suda.edu.cn;

[†]These authors contributed equally to this work.

METHODS

Chemicals

The following materials were acquired from Sigma-Aldrich Inc.: dimethyl sulfoxide (DMSO), N, N-dimethylformamide (DMF), 4-tert-butylpyridine (tbp), chlorobenzene (CB), lithium bis(trifluoromethylsulfonyl)imide (Li-TFSI), and gold (Au). $FAPbI_3$ and Spiro-OMeTAD were acquired from Advanced Election Technology Co., Ltd. L/D-p-FPhe molecules were procured from Shanghai Yuanye Bio-Technology Co., Ltd., while TCI Inc. provided buthaiaminium iodide (BAI).

Device Fabrication

PSCs were fabricated using an n-i-p structure: $FTO/SnO_2/FAPbI_3$/BAI/Spiro-OMeTAD/Au. The FTO substrates underwent 15 minutes of cleaning with deionized water, acetone, and ethanol. Then, the substrate was annealed for 30 minutes at 150 ℃ after being spin-coated with a SnO_2 nanoparticle film (1:4 by water) for 30 seconds at 3,000 rpm. 1 mL of a DMF/DMSO mixed solvent (v/v 4/1) was used to dissolve 1142.39 mg of $FAPbI_3$ and 36 mg of MACl to create the perovskite solution. The D/L-p-FPhe powders (1 mg/mL) were respectively dissolved in the precursor solutions to prepare two kinds of doping solutions. In the glove box, the $FAPbI_3$ layer was spin-coated for 30 seconds at 5000 rpm (800 μL of diethyl ether was injected at 15 s). Then, the samples were annealed for 10 minutes at 150 ℃. For the target samples, a designed frame with a NdFeB magnet placed on it to give a magnetic field of 50 mT, based on the previous studies focusing on the influence of the magnetic field to the perovskite film [1-3]. The direction of the magnetic field was confirmed by DFT simulations as shown in Tab. S5. Spiro-OMeTAD was spin coated for 40 seconds at 5000 rpm (90 mg in 1 mL CB). Finally, the samples were transferred into a thermal evaporation chamber (Suzhou Fangsheng FS380-S12) for the deposition of an Au electrode (100 nm) at a pressure < 2×10^{-6} Torr.

Characterizations:

J-*V* characteristics of the PSCs (0.09 cm^2) were taken using a Keithley 2400 source meter under a simulated AM 1.5G spectrum (Enli Technology Co., Ltd). Thin mask was used and performed anti-reflection treatment on devices (blackening). Utilizing an integrated system (Enlitech, Taiwan), EQEs were measured. The EL output characteristics of PSCs were collected using a Photo Research PR655 spectrometer and a Keithley 2400 source meter.

UV–vis absorption spectra were acquired using a Hitachi U-4100 spectrophotometer. GIWAXS measurements were carried out at the BL14B1 beamline of the China Shanghai Synchrotron Radiation Facility (SSRF). The X-ray used possessed a wavelength of 1.24 Å at a grazing incidence angle of 0.3° and energy of 10 KeV. Two-dimensional (2D) GIWAXS patterns were collected from a MarCCD 225 detector.UV photoelectron spectroscopy (UPS) was used to measure the work functions of samples. Using PicoHarp 300, which has time-correlated single-photon counting capabilities, time-resolved PL decay profiles were acquired. Steady-state photoluminescence (PL) measurements were carried out using a Horiba Jobin Yvon system. A thermal-electrically cooled CCD (Princeton Instruments, PIX-256E) recorded the microarea photoluminescence (μ-PL) spectra, which were obtained using a handmade optical microscope equipped with a Princeton Instrument grating spectrometer (ARC-SP-2356) for light coupling. An inverted microscope (Olympus, BX43) was used to get the PL microscopy images.

Experimental details

Supplementary Note 1. DFT Calculations.

We performed first-principles calculations based on density functional theory (DFT) by using a plane-wave basis set and the projected augmented wave method, as implemented in the VASP program [4,5]. The Perdew−Burke−Ernzerhof (PBE) functional with the generalized-gradient approximation (GGA) is used for the exchange-correlation functional in all geometry optimization and self-consistent field calculations [6,7]. A 4 × 4 × 1 gamma-centered k-mesh and a plane wave basis with a 400 eV cut-off energy are used for the geometry optimizations. We first relaxed the atomic positions and cell volumes using a conjugate gradient algorithm until all residual forces were smaller than 0.02 eV/Å. Continuation single-point energy calculations on the optimized geometries were performed to create the charge density difference graphs. We insert a vacuum slab with a thickness of 10−15 Å between the periodic slab-molecule structure along the z direction. The effect of the magnetic field is simulated by aligning all spin moments to a specific direction via the constrained spin method [8]. Interaction energies between molecules and perovskite slabs were computed using the same level of theory and using the following equation:

$$\Delta E_{int} = E_{mol+pvsk} - (E_{mol} + E_{pvsk})$$

where $E_{mol+pvsk}$, E_{mol}, and E_{pvsk} are the energies of the molecule/perovskite complex, only molecule, and only perovskite surface, respectively.

Supplementary Note 2. Photocurrent Measurements.

The photocurrent measurements were performed based on the Confocal Raman Imaging System (WITEC Alpha300 R). The excitation light source was a 633 nm laser with a power of 2 mW, which focused on the samples through an objective lens (Zeiss EC Epi-plan, 10× magnification, numerical aperture: 0.25). The photocurrent of the samples, at a bias of 0 V, was amplified by a low-noise current amplifier (FEMTO DLPCA-200), which also converted the photocurrent to voltage signal. During the photocurrent mapping, the laser spot was fixed and the sample stage scanned in a range of 1600×500 μm^2 with a minimal step size of 2 μm. The model used to fit the results is exponential decay, with the formula presented to be

$$y = A_1 * exp(-\frac{x}{L})$$

Supplementary Note 3. $V_{OC,loss}$ Measurement.

EQEs were collected by a QE-R quantum efficiency system of Enli Technology Co., Ltd. A National Renewable Energy Laboratory calibrated silicon solar cell was used to obtain the AM 1.5G solar simulator's light intensity. The system includes xenon lamp, monochromator, chopper, lock-in amplifier and calibrated silicon photodetector. The devices were shielded with a shading mask with an aperture area of 0.104 cm^2, and the beam spot area is 1*1 mm^2.

The EL spectra and electrical output characteristics of PSCs were collected under ambient condition using a Keithley 2400 source meter and a Photo Research spectrometer PR655.

Caculation of V_{oc} loss

According to Rau's work, the V_{oc} of a solar cell can be described as the equation listed below [9,10]

$$V_{OC} = \frac{k_B T}{q} ln(\frac{J_{SC}}{J_0})$$

where q is element charge, k_B is Boltzmann constant, T is temperature, J_{SC} is short-circuit current, J_0 is dark saturation current.

The voltage losses can be categorized into three contributions [11,12]:

$$\Delta V = \frac{E_g}{q} - V_{oc} = \left(\frac{E_g}{q} - V_{OC}^{SQ}\right) + \left(V_{OC}^{SQ} - V_{OC}^{rad}\right) + (V_{OC}^{rad} - V_{OC})$$

V_{OC}^{SQ} in the equation is the maximum voltage based on the Shockley-Queisser (SQ) limit, where solar cells are recognized to be ideal without absorption below the gap. E_g is the bandgap, V_{OC}^{rad} is the V_{oc} where only radiative recombination occurred in PSCs.

In the SQ limit, V_{OC}^{SQ} follows [13]:

$$V_{OC}^{SQ} = \frac{k_B T}{q} ln\left(\frac{J_{SC}^{SQ}}{J_0^{SQ}} + 1\right) = \frac{k_B T}{q} ln(\frac{q\int_{E_g}^{\infty} \emptyset_{AM1.5}(E) d_E}{q\int_{E_g}^{\infty} \emptyset_{BB}(E) d_E} + 1)$$

$$\emptyset_{BB} = \frac{2\pi E^2}{h^3 c^2} \frac{1}{exp\left(\frac{E}{k_B T}\right) - 1}$$

J_{SC}^{SQ} and J_0^{SQ} are J_{SC} and J_0 in S-Q limit respectively, $\emptyset_{AM1.5}$ is solar cell radiative spectrum, $\emptyset_{BB}$ is black-body radiative spectrum, c is light speed in vacuum.

The first V_{oc} loss component is due to the non-ideal EQE_{PV},

$$\Delta V_1 = \frac{E_g}{q} - V_{OC}^{SQ}$$

The second V_{oc} loss component originates from the energy loss related to additional thermal radiation of solar cell in dark. The difference between V_{OC}^{SQ} and V_{OC}^{rad} is caused by the non-step function like EQE_{PV} of the real-world devices.

$$V_{OC}^{rad} = \frac{k_B T}{q} ln\left(\frac{J_{SC}^{rad}}{J_0^{rad}} + 1\right) = \frac{k_B T}{q} ln\left(\frac{q\int_{E_g}^{\infty} EQE_{PV}\emptyset_{AM1.5}(E)d_E}{q\int_{E_g}^{\infty} EQE_{PV}\emptyset_{BB}\,(E)d_E} + 1\right)$$

$$\Delta V_2 = V_{OC}^{SQ} - V_{OC}^{rad}$$

The third V_{oc} loss component is associated with the non-radiative recombination in devices, that can be calculated as:

$$\Delta V_{OC}^{nonrad} = \frac{k_B T}{q} ln\left(\frac{J_{SC}}{J_0^{rad}}\right) - V_{OC}$$

Rau's reciprocity relation is here expressed in terms of the dark saturation current J_0 and the absolute quantum efficiency of electroluminescence [14]:

$$J_0^{rad} = EQE_{EL} \cdot J_0$$

$$\Delta V_3 = \Delta V_{OC}^{nonrad} = \frac{k_B T}{q} ln\left(\frac{J_{SC}}{EQE_{EL} \cdot J_0}\right) - \frac{k_B T}{q} ln\left(\frac{J_{SC}}{J_0}\right) = -\frac{k_B T}{q}(lnEQE_{EL})$$

Supplementary Note 4. The evaluation of the trap density of the perovskite.

***t*DOS measurement**: For *C*-*V* measurement, the DC bias was from 0 V to the V_{OC} for the cells. For the TAS measurement, the DC bias was fixed at 0 V and the amplitude of the AC bias was 20 mV. The scanning range of the AC frequency was 1K -10 MHz. The *t*DOS (N_T (E_ω)) was calculated by using equation

$$N_T(E_w) = -\frac{1}{qkT}\frac{wdC}{dw}\frac{V_{bi}}{W}$$

where V_{bi} is the build-in potential of devices and W is the depletion width, derived from the Mott-Schottky analysis of the *C*-*V* measurement. *k*, *q*, *T*, *ω,* and *C* are specific capacitance, Boltzmann's constant, temperature angular, frequency and elementary charge, respectively. The demarcation energy is;

$$E_w = kTln(\frac{w_0}{w})$$

where ω_0 is the attempt-to-escape angular frequency;

$$\omega_0 = 2\pi v_0 T^2$$

where v_0 is the temperature-independent attempt-to-escape frequency, derived from the temperature-dependent *C*-*f* measurements. All the measurements were taken under dark condition.

DCLP measurement: The DLCP method uses a variable δV (e.g., 20 to 200 mV) to measure the junction capacitance and acquire the capacitance contribution from the trap states by taking advantage of the information embedded in the higher order terms. With the determination of C_0 and C_1, the carrier density (*N*) that includes both free carrier density (N_0) and trap density (N_T) at a certain position X from the junction barrier was calculated by [15]:

$$N = -\frac{C_0^3}{2q\varepsilon A^2 C_1}$$

where ε=6.5 is the dielectric constant of perovskite, *q* is the elementary charge, A=0.16 cm^2 is the active area of the cells. The trap density of samples was estimated by subtracting the estimated free carrier density. The free carrier density is estimated by measuring the carrier density at a high AC frequency when the total carrier density tends to saturate with the further increase of the AC

frequency. The trap density at a much lower AC frequency (large E_{ω}) can be estimated by subtracting the estimated free carrier density from the total carrier density measured at the low AC frequency. The profiling distance from the junction barrier is given by $\varepsilon A/C_0$. [16] The specific parameters were determined according to previous reports [17,18].

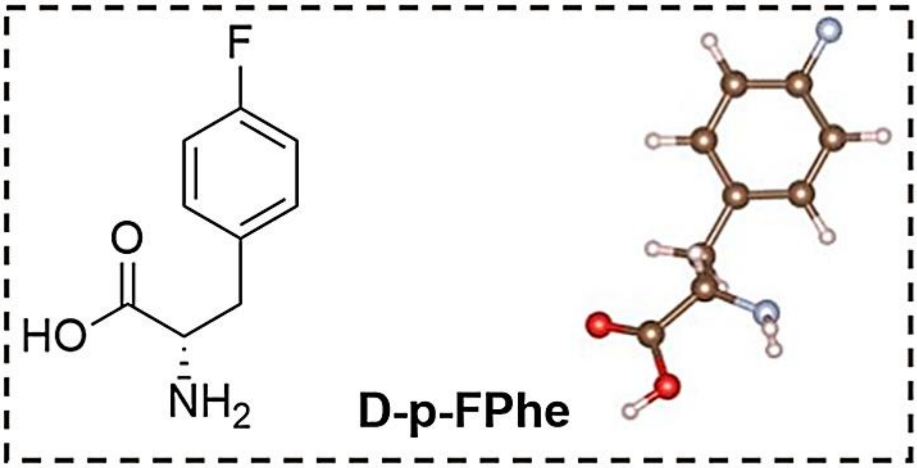

Figure S1. Molecular formula and DFT results for the net magnetization of D-p-Fluorophenylalanine molecule. The right diagram corresponds to the DFT results, and the chiral molecules lack any magnetic dipole moments, so there is no mark.

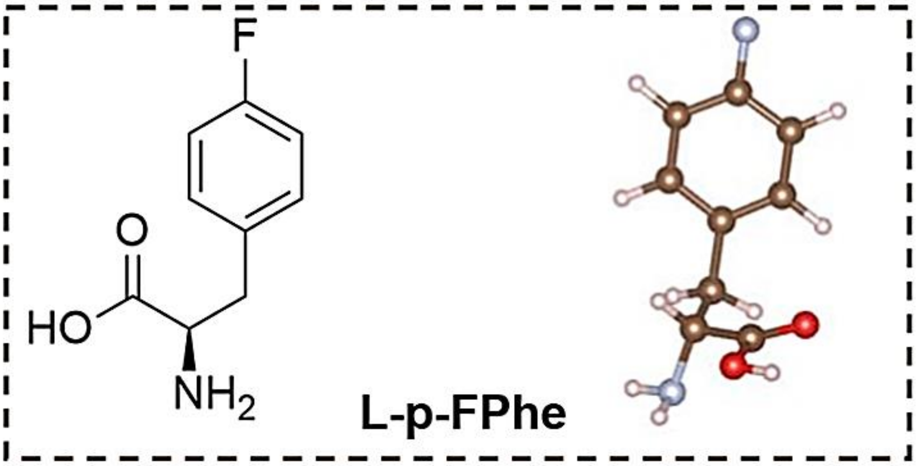

Figure S2. Molecular formula and DFT results for the net magnetization of L-p-Fluorophenylalanine molecule. The right diagram corresponds to the DFT results, and the complete chiral molecule lacks any magnetic dipole moments, so there is no mark.

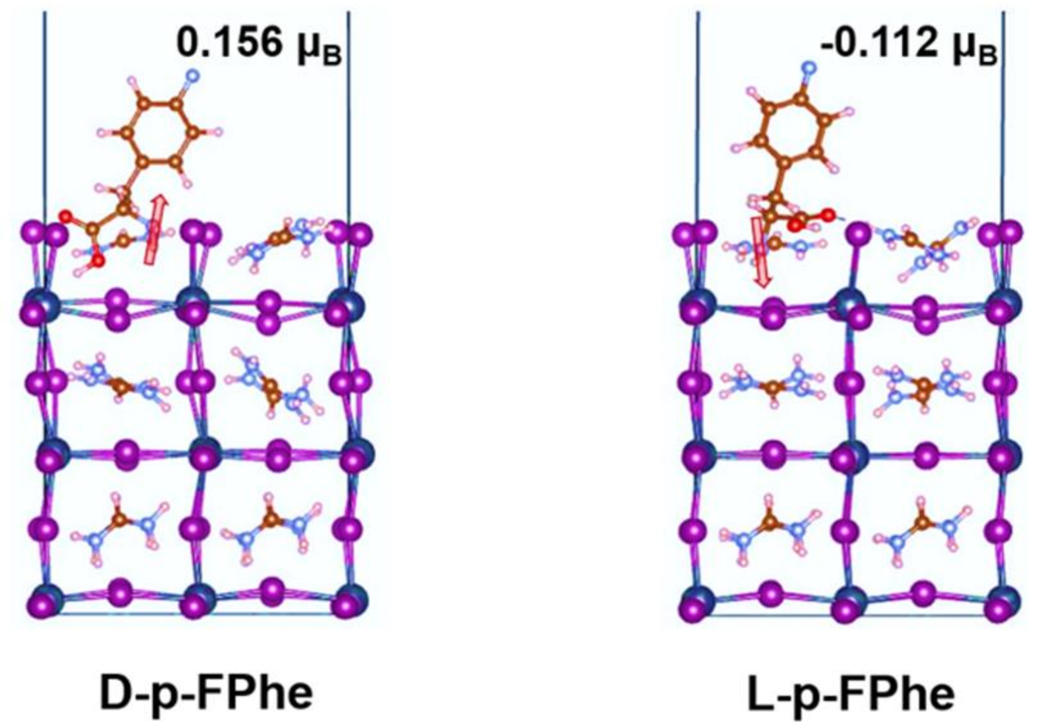

Figure S3. DFT results of the net magnetization when the p-Fluorophenylalanines are introduced to the $FAPbI_3$ cage, and the net magnetization mainly originated by N ion of the alanine. The result revealed the presence of magnetic dipole moments between chiral molecules and FA ions, with values of 0.156 μ_B and -0.112 μ_B (μ_B representing the Bohr magneton) along the z-axis for D/L-p-fluorophenylalanine (D/L-p-FPhe) molecules.

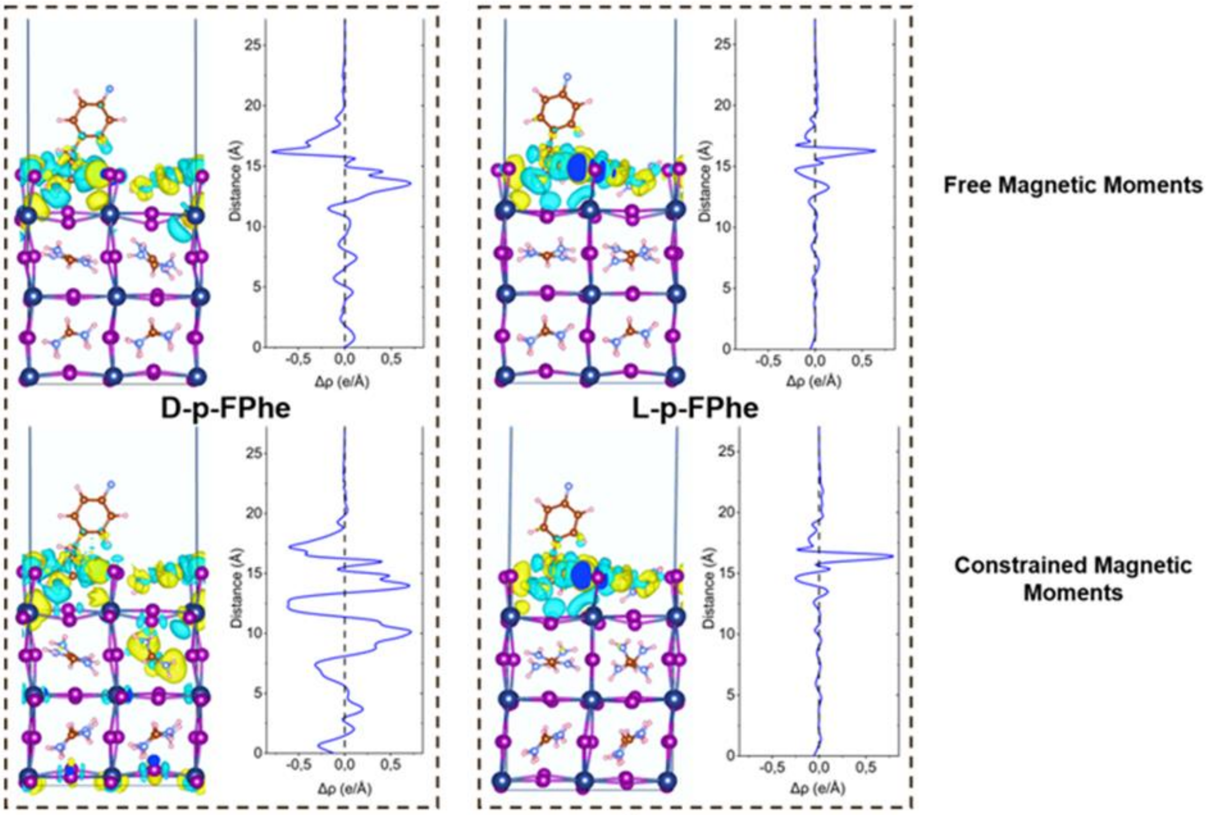

Figure S4. The charge density profiles for the D/L-p-FPhe molecules on the perovskite surface with or without magnetic field based on CCD calculations. Specifically, all spin magnetic moments were restricted in the molecule/perovskite system to align along the *z*-axis direction.

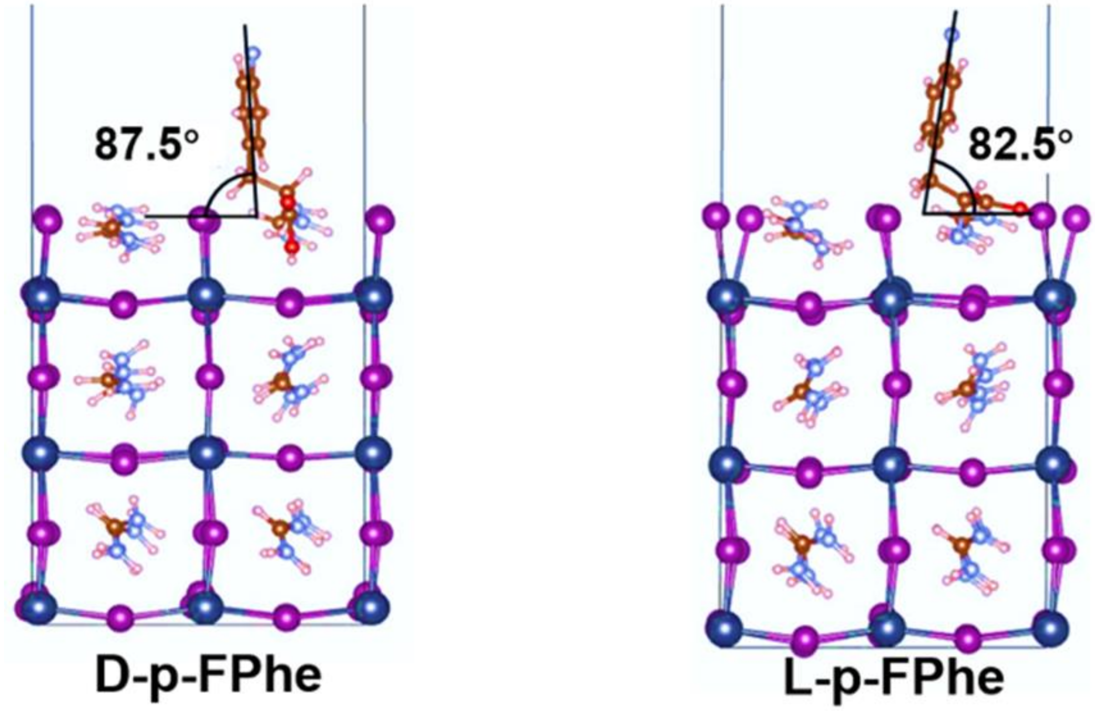

Figure S5. DFT results of the angles when the p-Fluorophenylalanines are introduced to the $FAPbI_3$ cage.

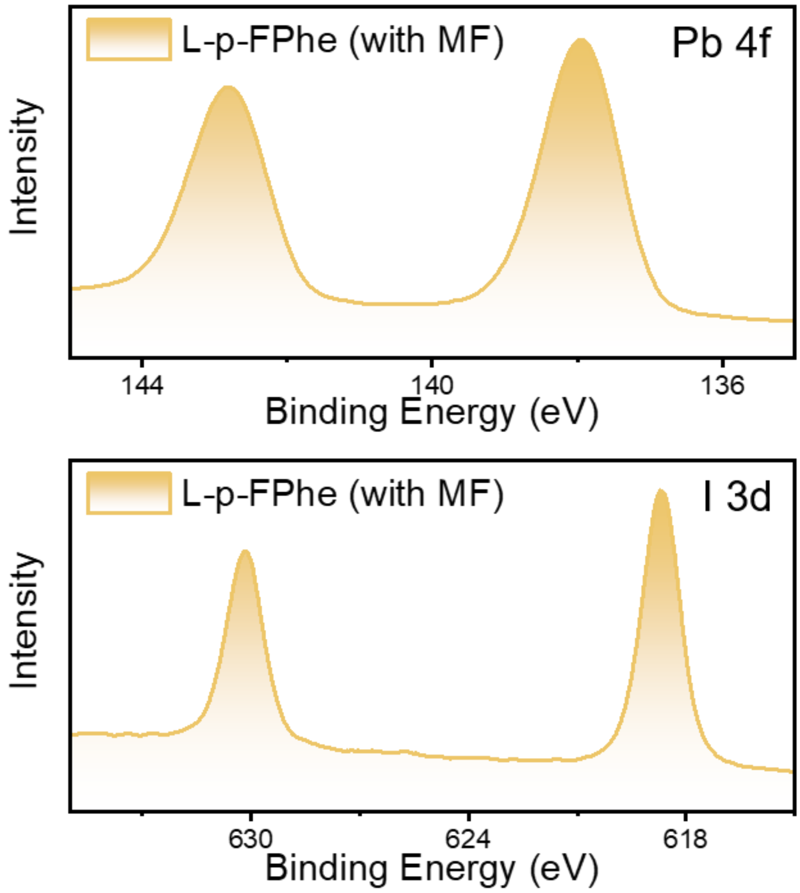

Figure S6. XPS spectra of perovskite film tread with L-p-FPhe under magnetic field and control $FAPbI_3$ perovskite film.

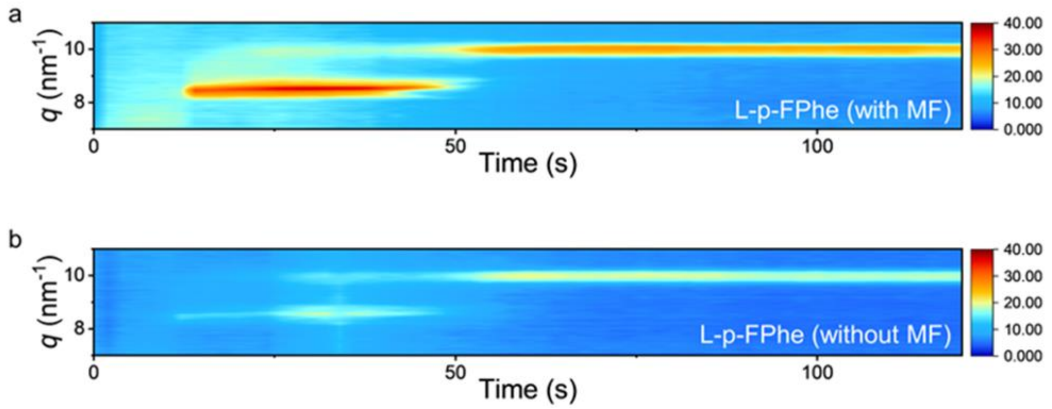

Figure S7. 2D plots of the azimuthally integrated scattering intensity varies with annealing time along the ring at q = 10 nm^{-1} for the L-p-FPhe doping samples with/without magnetic field. To compare the outcomes of these two effects, L-p-FPhe based samples were fabricated, which were demonstrated to solely possess passivation effects without exhibiting magnetic dipole moments.

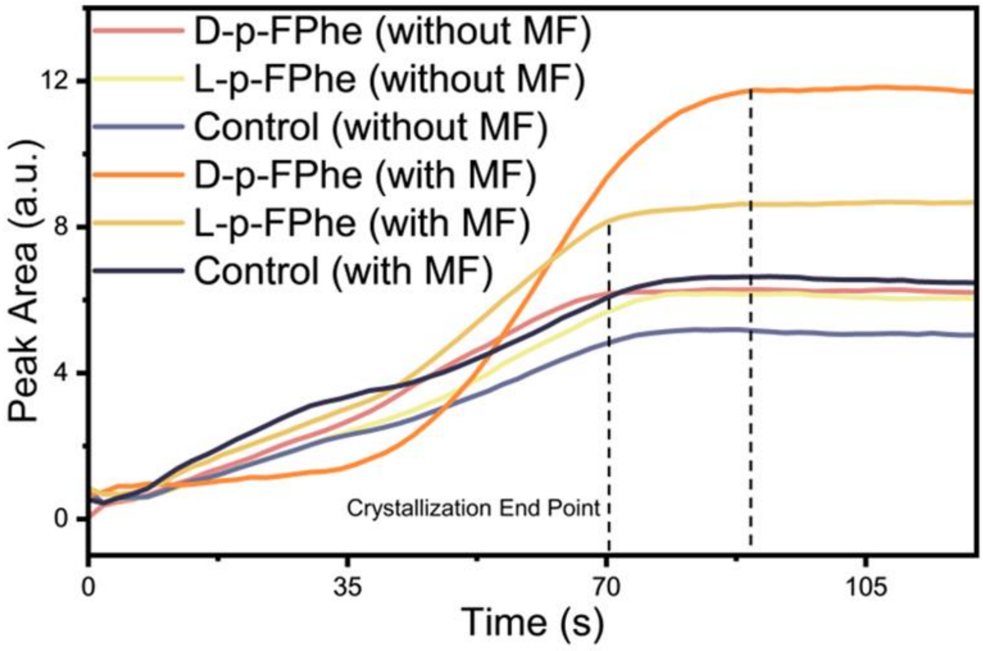

Figure S8. Corresponding variations in the (001) peak intensity with time during $FAPbI_3$ film growth under annealing conditions.

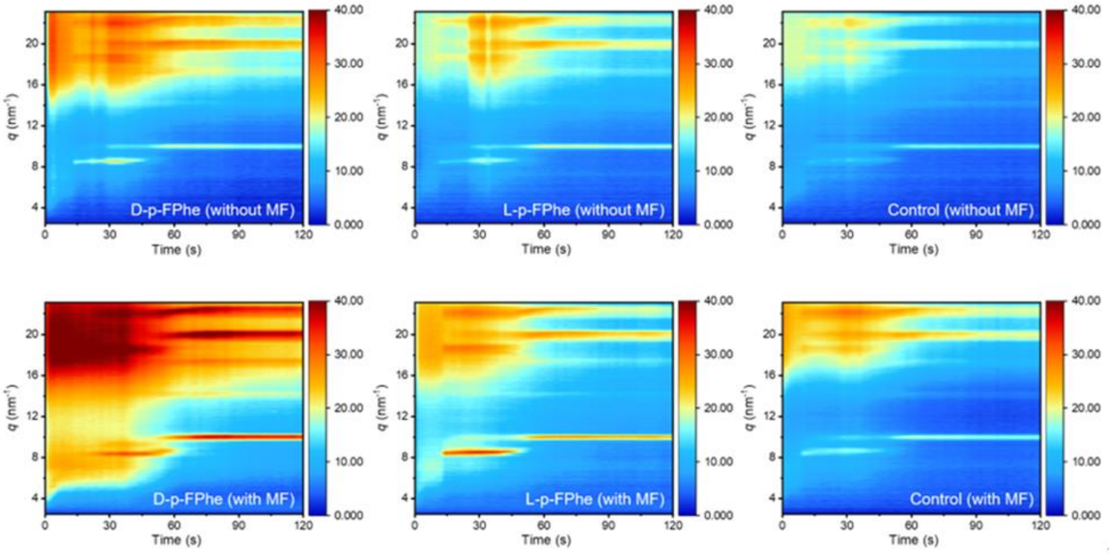

Figure S9. In situ GIWAXS monitoring the crystal growth process of D/L-p-FPhe and control perovskite films covering q of 0-24 nm^{-1}. Top view image of $FAPbI_3$ (001) peaks in GIWAXS images. Expanding the analysis beyond the core (001) crystal face, the GIWAXS results encompassing multiple crystal faces within a broader range consistently yielded similar outcomes.

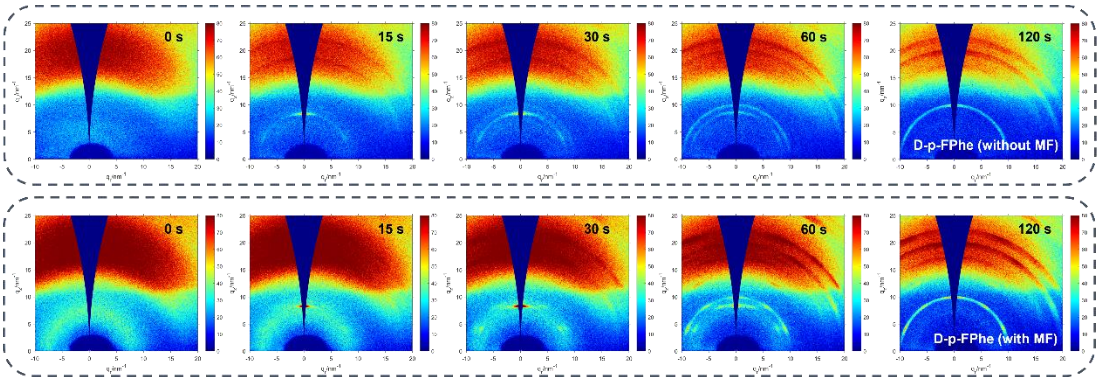

Figure S10. 2D-GIWAXS patterns of the D-p-FPhe doped perovskite films with/without magnetic field during spin-coating time, before and after anti-solvent dropping, annealing for 30, 60 and 120 s.

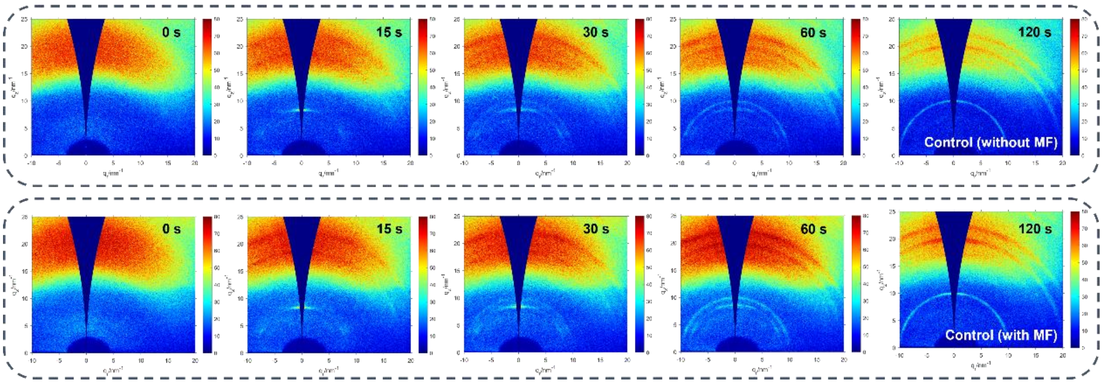

Figure S11. 2D-GIWAXS patterns of the perovskite films with/without magnetic field during the spin-coating time, before and after anti-solvent dropping, annealing for 30, 60 and 120 s.

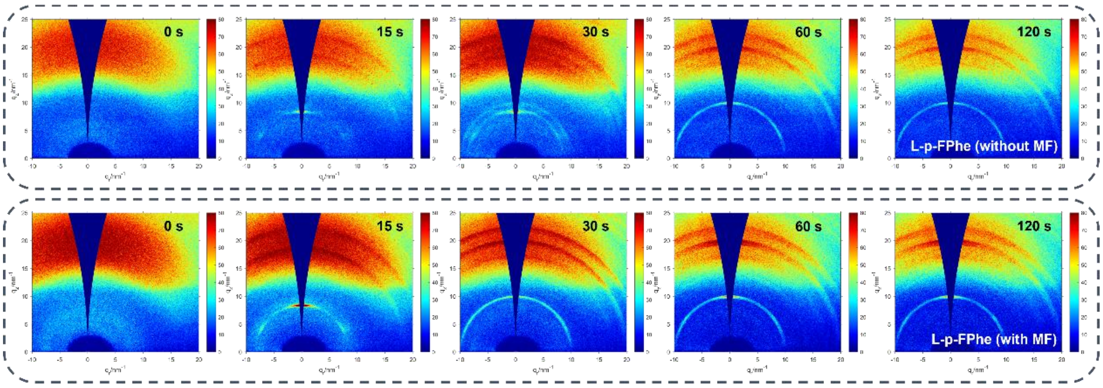

Figure S12. 2D-GIWAXS patterns of the L-p-FPhe doped perovskite films with/without magnetic field during spin-coating time, before and after anti-solvent dropping, annealing for 30, 60 and 120 s.

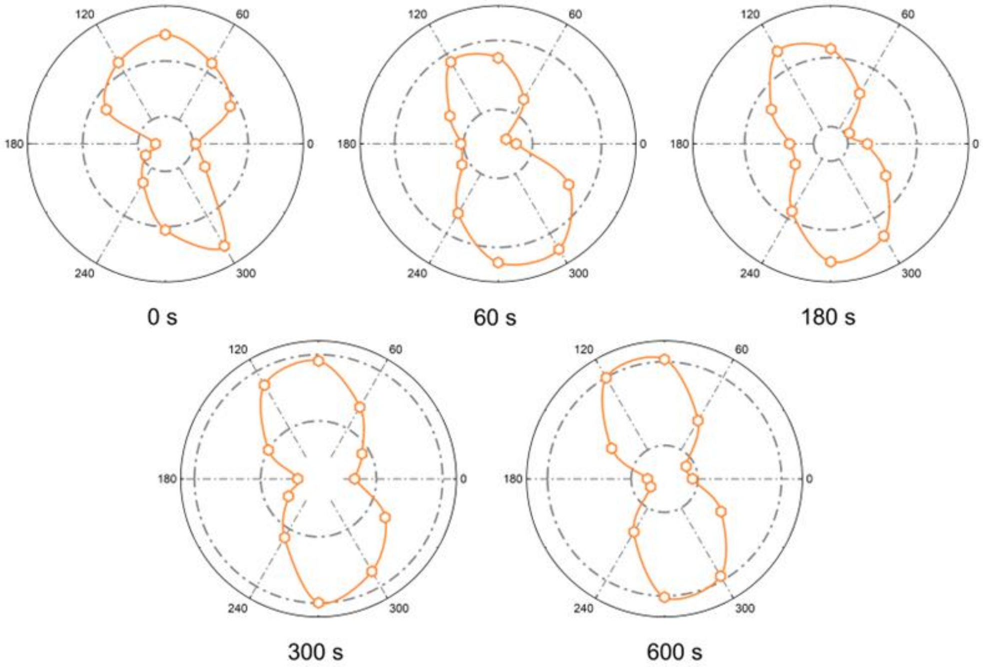

Figure S13. Polar images of the target samples with annealing time of 0, 60, 180, 300, 600 s under the polarized incident light.

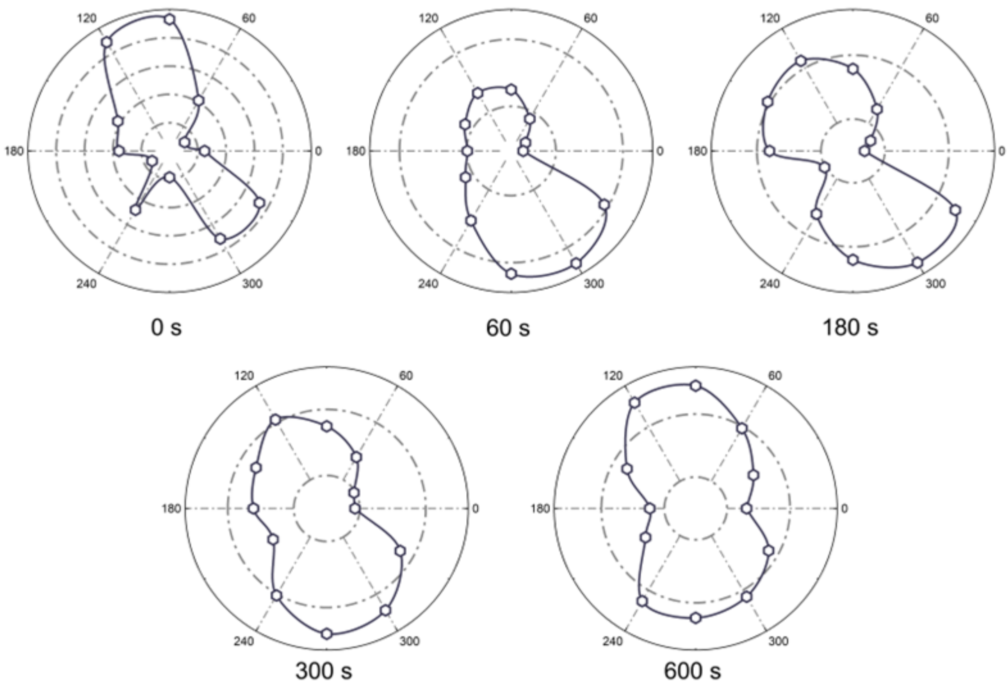

Figure S14. Polar images of the control samples with annealing time of 0, 60, 180, 300, 600 s under the polarized incident light.

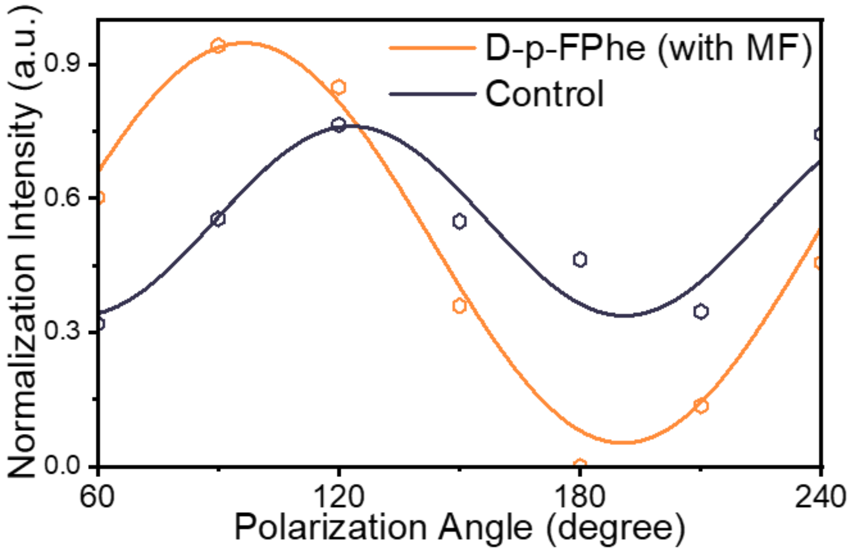

Figure S15. Normalized emission intensities as a function of detection polarization angle (ϕ) for the final target sample and control sample. Solid curves are fits to cos2 ϕ.

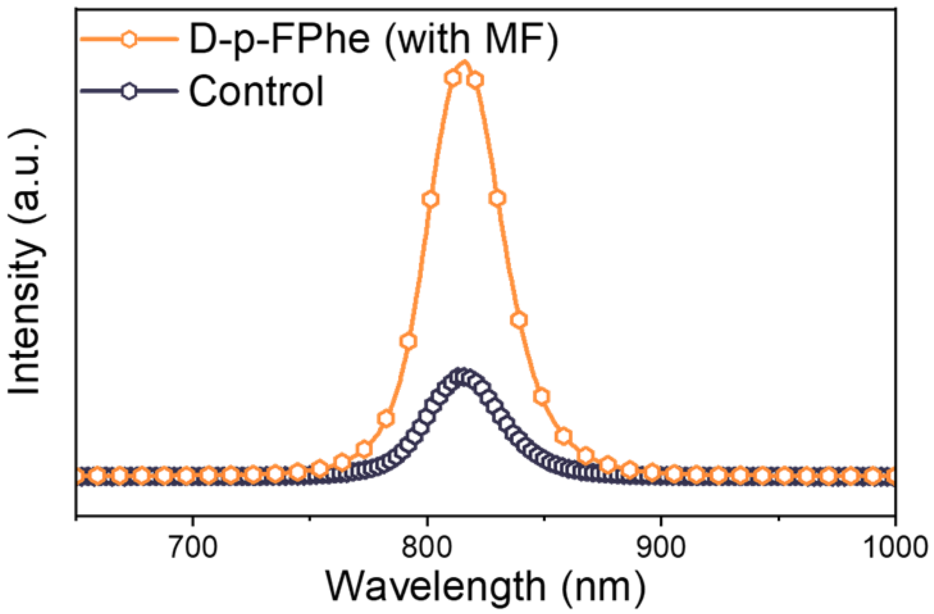

Figure S16. Steady state PL spectra of target and control films.

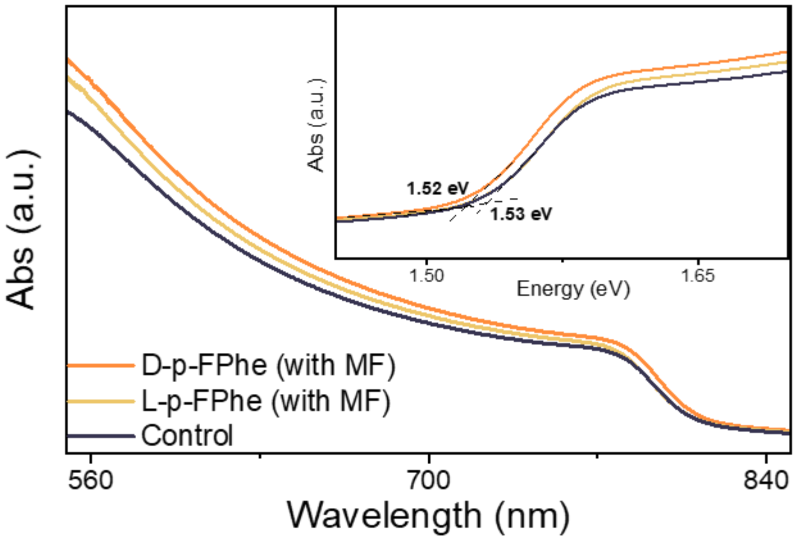

Figure S17. Absorption spectra of target and control films.

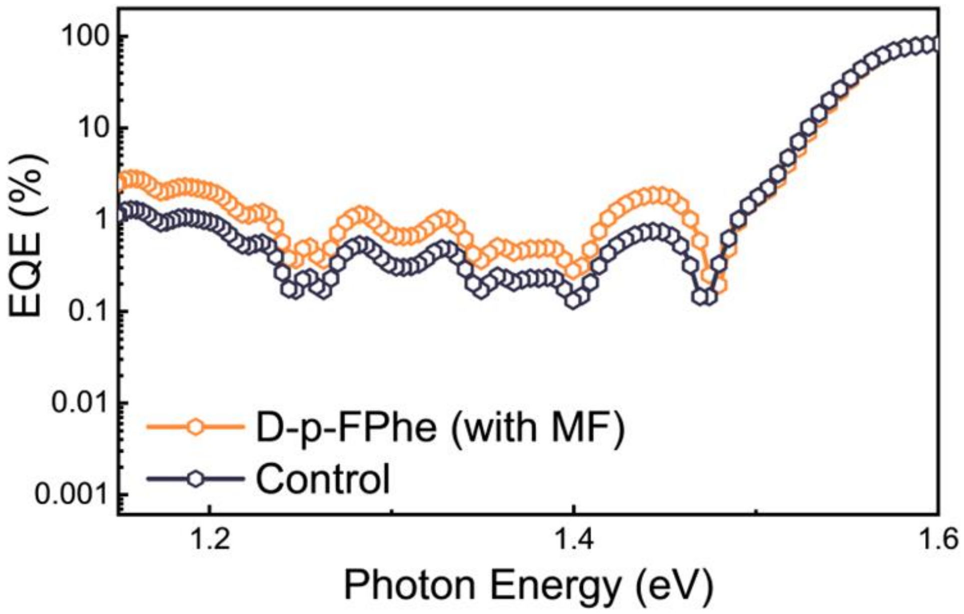

Figure S18. EQE of D-p-FPhe (with MF) and control devices.

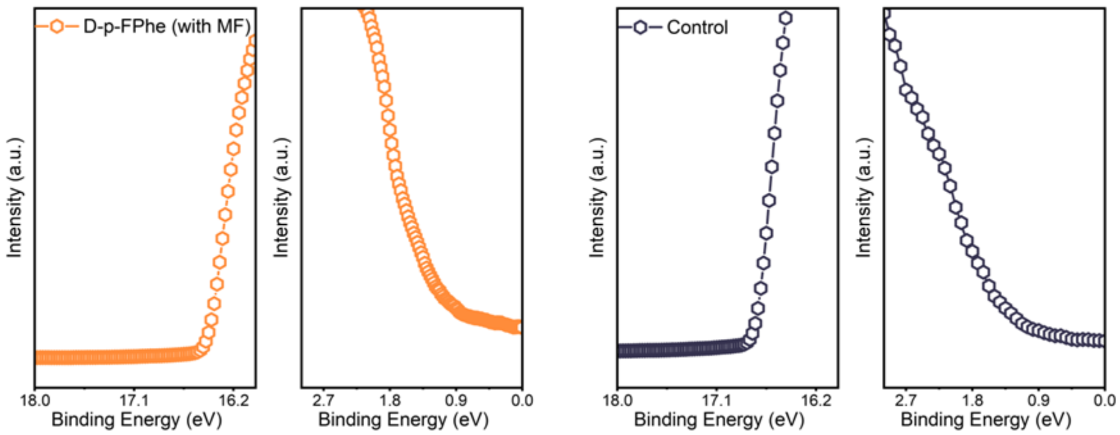

Figure S19. UPS spectra of target and control films and the schematic illustration of the corresponding energy band alignment of perovskite solar cells.

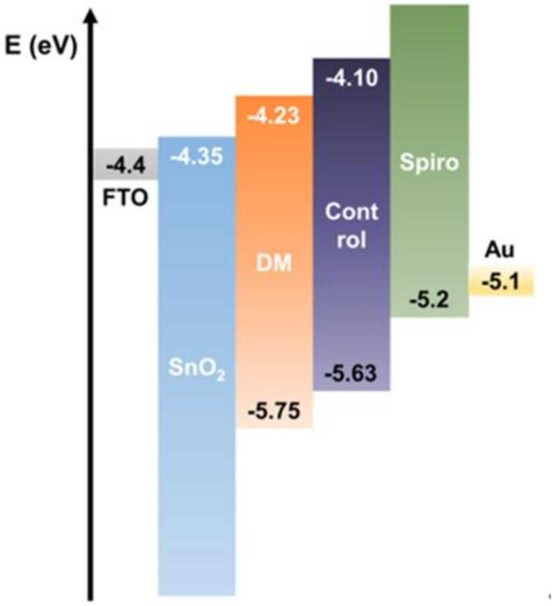

Figure S20. Schematic illustration of the corresponding energy band alignment of perovskite solar cells.

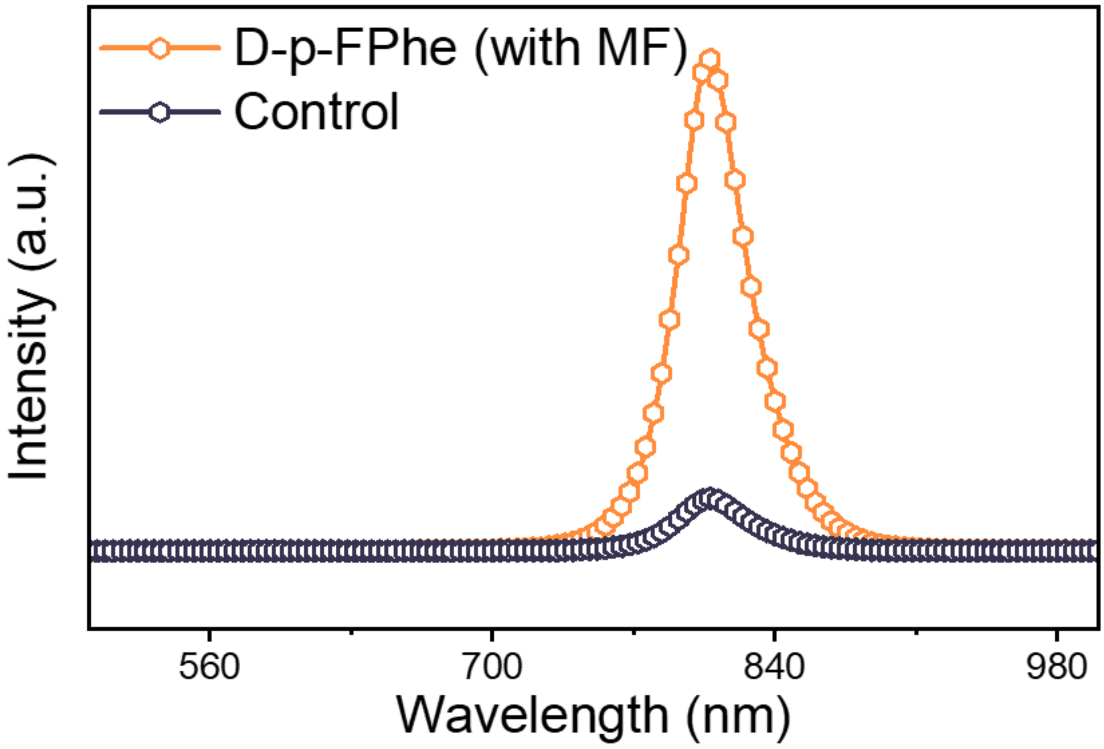

Figure S21. EL curves of the D-p-FPhe (with MF) and control devices.

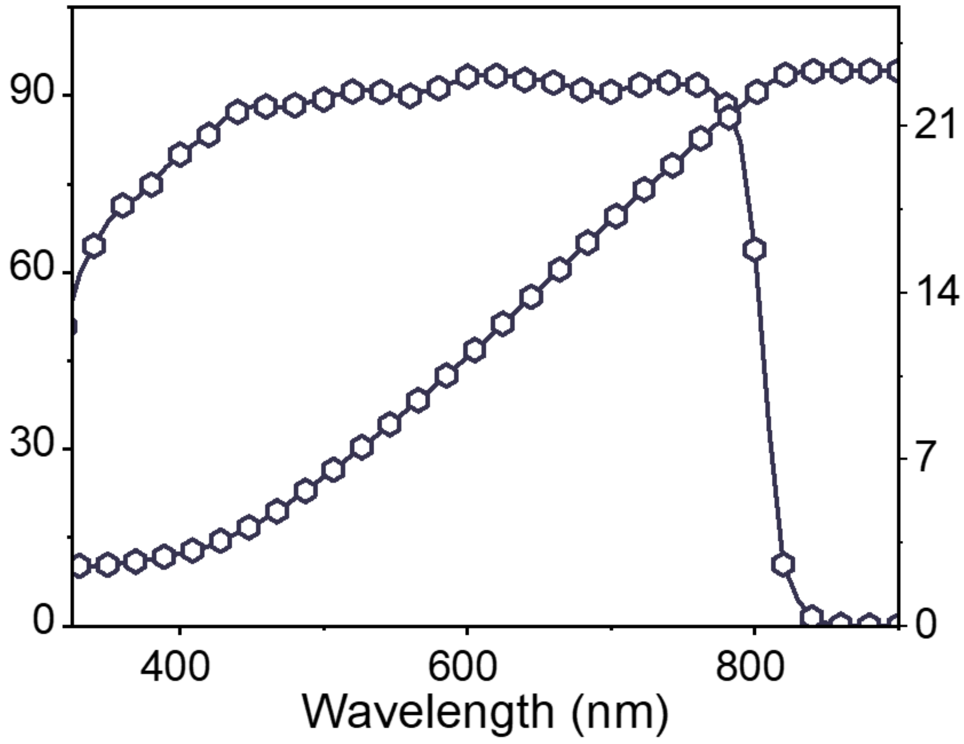

Figure S22. EQE diagram and corresponding integral current of the control device.

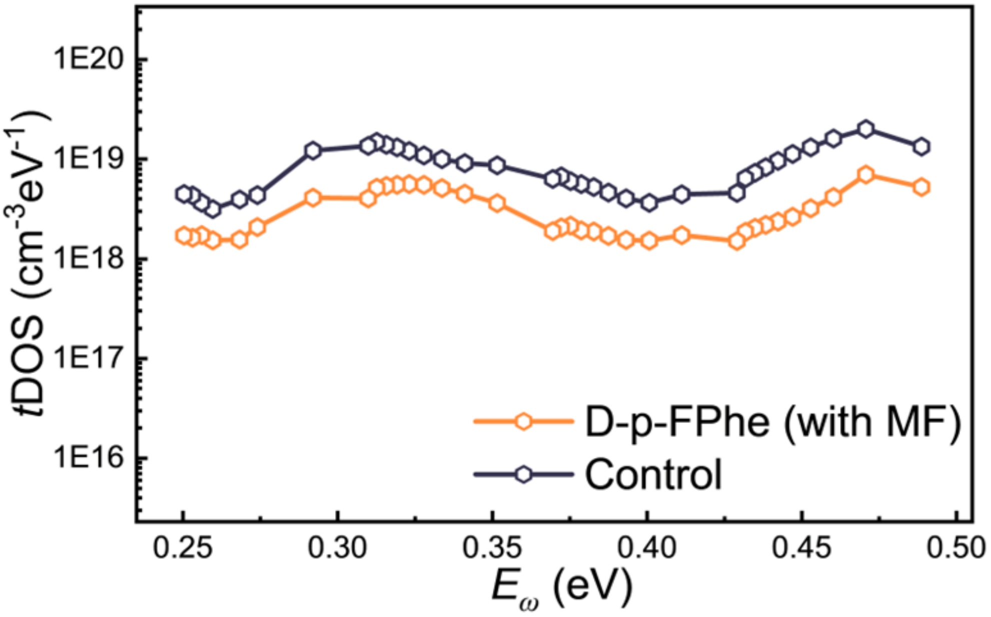

Figure S23. *t*DOS curves of D-p-FPhe (with MF) and control devices.

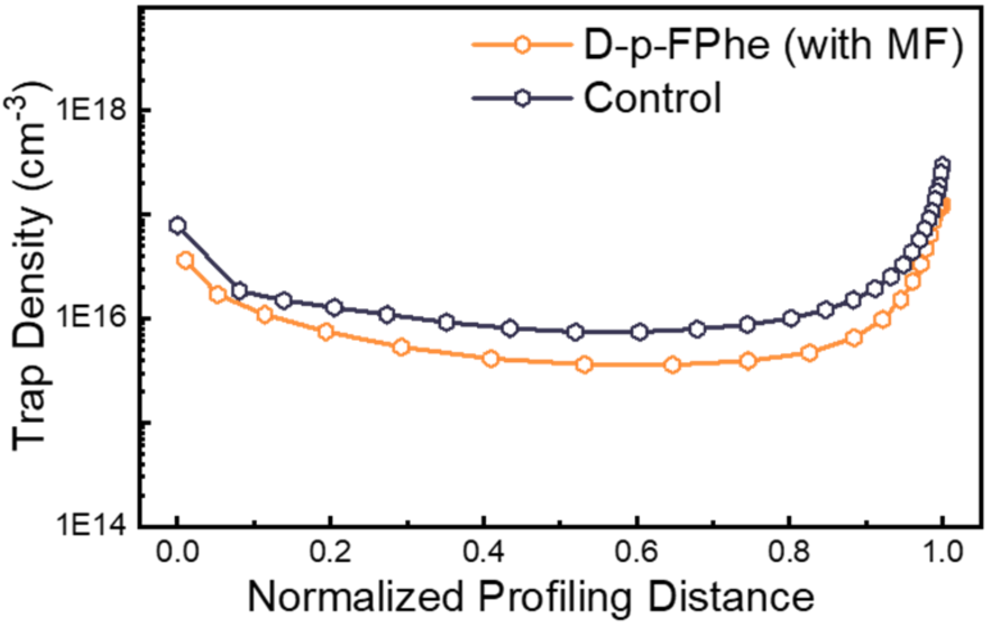

Figure S24. DLCP curves of the D-p-FPhe (with MF) and control devices.

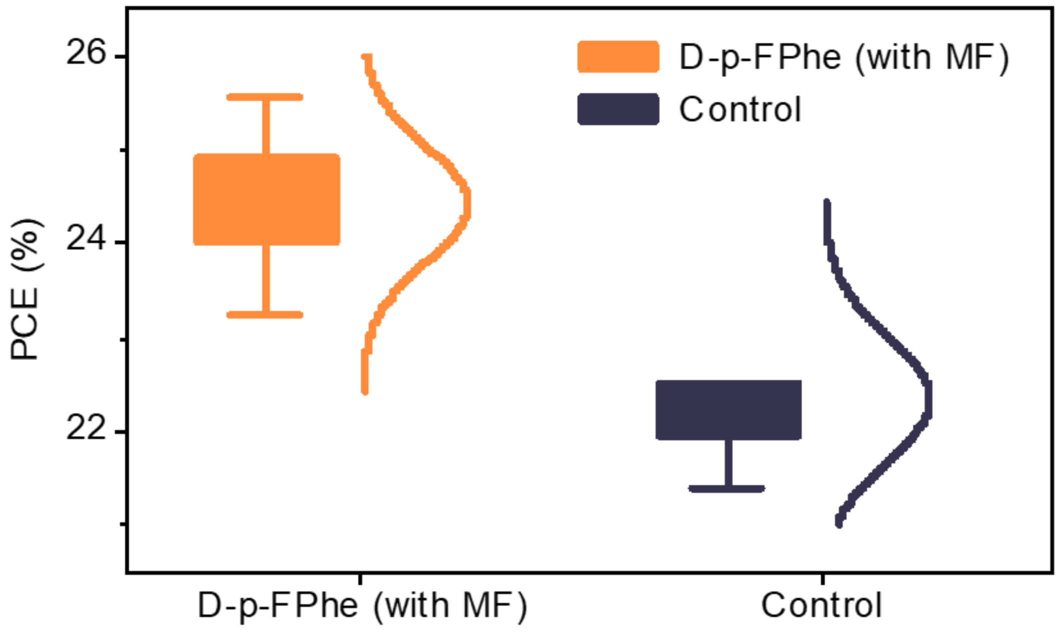

Figure S25. Device efficiency statistics of 32 D-p-FPhe (with MF) and control devices, respectively.

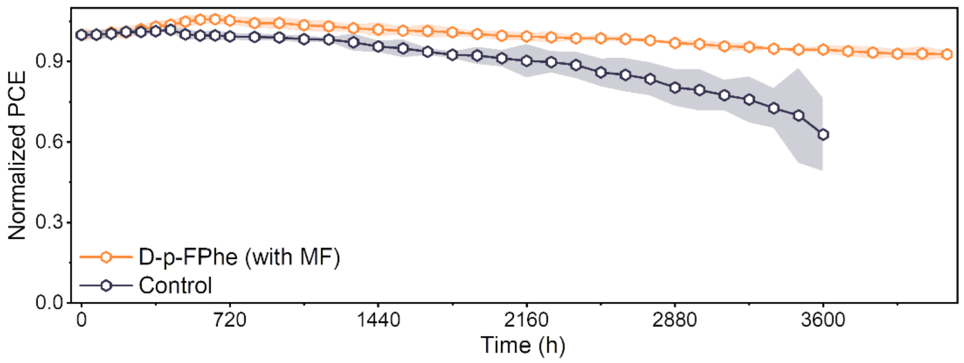

Figure S26. Evolution of PCE relative to the initial PCE for the D-p-FPhe-doped devices with magnetic field application, L-p-FPhe-doped devices without magnetic field application and $FAPbI_3$ devices over 4200 hours of storage in dry air. Each average (symbol) and standard deviation (error bar) was calculated from six solar cells.

Table S1. Carrier lifetime of the D-p-FPhe (with MF) and control films extracted from TRPL measurements.

	$<\tau>$	$<\tau_1>$	$<\tau_2>$
D-p-Fphe (with MF)	3.60	0.22	4.80
Control	0.42	0.03	0.63

Table S2. Fitting parameters of the formula for photocurrent measurement of the D-p-FPhe (with MF) and control devices.

Device	D-p-Fphe (with MF)	Control
Y_0	-1.97 ±0.02	0.63±0.00
A_1	7.95±0.03	3.10 ±0.01
l	325.46± 3.11 μm	230.34 ± 1.79 μm
Reduced Chi-	0.04	0.004
R^2	0.99	0.99
R^2 after regulation	0.99	0.99

Table S3. Performance factors of the D-p-FPhe (with MF) and control devices.

	V_{oc} (V)	J_{sc} (mA/cm^2)	FF (%)	PCE (%)
D-p-FPhe (with MF) （Reverse）	1.16	26.05	83.46	25.22
Control (Reverse)	1.12	25.95	81.53	23.72
D-p-FPhe (with MF) (Forward)	1.15	25.99	81.10	24.25
Control (Forward)	1.10	25.98	78.09	22.22

Table S4. V_{oc} loss analysis of the D-p-FPhe (with MF) and control devices.

	Real-V_{oc} (V)	V_{OC}^{SQ} (V)	ΔV_1 (mV)	ΔV_2 (mV)	ΔV_3 (mV)	Total V_{loss} (mV)	Calculated V_{OC} (V)
D-p-Fphe (with MF)	1.16	1.252	268	5.44	86.26	359.70	1.16
Control	1.12	1.261	269	16.89	126.82	412.71	1.12

Table S5. Binding energy of the chiral molecules with the perovskites in different directions of the magnetic field. A same direction of the magnetic field and the magnetic moment should be confirmed for D-p-FPhe while L-p-FPhe presented similar in two directions.

	Free Magnetic Field (eV)	Constrained along +z direction (eV)	Constrained along -z direction (eV)
D-p-FPhe	-1.45	-7.08	-1.41
L-p-FPhe	-1.42	-1.47	-1.46

References

1. Wang H, Lei J and Gao F *et al.* Magnetic Field-Assisted Perovskite Film Preparation for Enhanced Performance of Solar Cells. *ACS Appl Mater Interfaces* 2017; **9**: 21756-62.
2. Corpus-Mendoza AN, Moreno-Romero P and Hu H. Impact of magnetic fields on the morphology of hybrid perovskite films for solar cells. *AIP Adv* 2018; **8**: 05522.
3. Corpus-Mendoza AN, Cruz-Silva BS and Ramirez-Zúñiga G *et al.* Use of Magnetic Fields for Surface Modification of PbI2 Layers to Increase the Performance of Hybrid Perovskite Solar Cells. *J Electron Mater* 2020; **49**: 3106–13.
4. Kresse G, Hafner J. Ab initio molecular dynamics for liquid metals. *Phys Rev B* 1993; **47**: 558.
5. Kresse G, Furthmüller J. Efficiency of ab initio total energy calculations for metals and semiconductors using a plane-wave basis set. *Comput Mater Sci* 1996; **6**: 15−50.
6. Komsa HP, Broqvist P Pasquarello. Alignment of defect levels and band edges through hybrid functionals: Effect of screening in the exchange term. *Phys Rev B* 2010; **81**: 205118.
7. Freysoldt C, Lange B and Neugebauer J *et al.* Electron and chemical reservoir corrections for point-defect formation energies. *Phys Rev B* 2016; **93**: 165206.
8. Deger C. Strain-enhanced dzyaloshinskii–moriya interaction at Co/Pt interfaces. *Sci Rep* 2020; **10**: 12314.
9. Rau U. Reciprocity relation between photovoltaic quantum efficiency and electroluminescent emission of solar cells. *Phys Rev B* 2007; **76**: 085303.
10. Vandewal K, Tvingstedt K and Gadisa A *et al.* Relating the open-circuit voltage to interface molecular properties of donor:acceptor bulk heterojunction solar cells. *Phys Rev B* 2010; **81**: 125204.
11. Yao JZ, Kirchartz T and Vezie MS *et al.* Quantifying Losses in Open-Circuit Voltage in Solution-Processable Solar Cells. *Phys Rev Appl* 2015; **4**: 014020.
12. Liu J, Chen SS and Qian D *et al.* Fast charge separation in a non-fullerene organic solar cell with a small driving force. *Nat Energy* 2016; **1**: 16089.

13. Li FZ, Deng X and Qi F *et al.* Regulating Surface Termination for Efficient Inverted Perovskite Solar Cells with Greater Than 23% Efficiency. *J Am Chem Soc* 2020; **142**: 20134-42.

14. Tvingstedt K, Malinkiewicz O and Baumann A *et al.* Radiative efficiency of lead iodide based perovskite solar cells. *Sci Rep* 2014; **4**: 6071.

15. Heath JT, Cohen JD and Shafarman WN. Bulk and metastable defects in $CuIn_{1-x}Ga_xSe_2$ thin films using drive-level capacitance profiling. *J Appl Phys* 2004; **95**: 1000-10.

16. Zhao JJ, Deng YH and Wei HT *et al.* Strained hybrid perovskite thin films and their impact on the intrinsic stability of perovskite solar cells. *Sci Adv* 2017; **3**: eaao5616.

17. Bleicher M and Lange E. Schottky-barrier capacitance measurements for deep level impurity determination. *Solid-State Electron* 1973; **16**: 375-80.

18. Ni ZY, Bao CX and Liu Y *et al.* Resolving spatial and energetic distributions of trap states in metal halide perovskite solar cells. Science 2020; **367**: 1352-8.
